# Proximity Labeling Reveals RNA-Binding Proteins Associating with the Human Mitochondrial Import Receptor TOMM20

**DOI:** 10.1021/acs.jproteome.5c00905

**Published:** 2025-12-22

**Authors:** Saira Akram, Katharina I. Zittlau, Karan Sharma, Julia C. Fitzgerald, Nisha Rafiq, Boris Maček, Ralf-Peter Jansen

**Affiliations:** † Interfaculty Institute of Biochemistry, 9188 University of Tübingen, 72076 Tübingen, Germany; ‡ Proteome Center Tübingen, University of Tübingen, 72076 Tübingen, Germany; § Department of Biomolecular Sciences, Weizmann Institute of Science, 76100 Rehovot, Israel; ∥ Department of Neurodegeneration, Hertie Institute for Clinical Brain Research, Centre for Neurology, Faculty of Medicine, University of Tübingen, 72076 Tübingen, Germany

**Keywords:** TOMM20, TOMM70, proximity labeling, APEX2, mitochondrial import, proteomics, SYNJ2BP

## Abstract

The import of most mitochondrial proteins requires that their precursor proteins be bound by the peripheral receptor proteins TOM20, TOM22, and TOM70. Budding yeast TOM20 and TOM70 have been extensively studied regarding their interaction partners and recognized substrates; however, little data is available for metazoan cells. Using APEX2-based proximity labeling, we created association profiles for human TOMM20 and TOMM70 in HeLa cells. We focused particularly on their interactions with RNA-binding proteins (RBPs) because there is evidence of RNA association with the mitochondrial outer membrane (MOM) and of local translation at the mitochondrial surface, however, these processes are poorly understood. Our results demonstrate that several RBPs and translation factors preferentially associate with TOMM20 rather than TOMM70. These include SYNJ2BP, a previously identified membrane-bound RBP that binds and protects mRNA encoding mitochondrial proteins. Inhibiting translation with puromycin increased the association of these RBPs with TOMM20 compared to TOMM70. This suggests that TOMM20, but not TOMM70, may play a role in maintaining cellular homeostasis during translation stress by retaining protective RBPs and translation-related proteins at the MOM.

## Graphical abstract

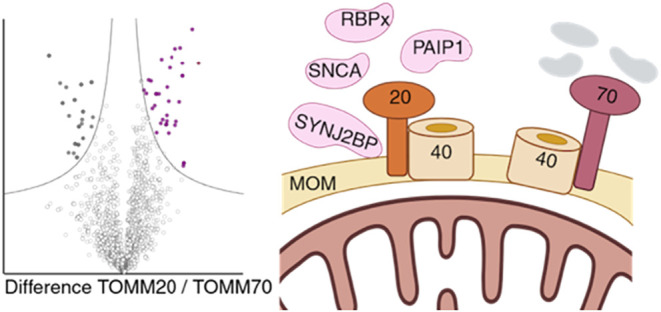

## Introduction

The process of protein sorting to intracellular organelles is an essential process in eukaryotic cells because most proteins are encoded in the nucleus but play a role in specific cellular compartments. Mitochondria must import and sort more than 1000 different preproteins to the various intramitochondrial compartments. These preproteins contain specific targeting signals that are recognized by the mitochondrial import machinery.^1^ One major entry gate is the translocase of the outer mitochondrial membrane (TOM). This protein complex contains the translocation pores formed by the TOM40 subunit (TOMM40 in mammals) and a central preprotein receptor subunit, TOM22/TOMM22. TOMM22 accepts its substrates from two additional receptor subunits, TOM20/TOMM20 and TOM70/TOMM70, and transfers them to the TOMM40 translocation pore. TOMM20 and TOMM70 recognize the targeting signals but they differ in substrate specificity, although overlap can be observed.^2,3^ TOMM20 binds to preproteins that are targeted to the mitochondrial inner membrane (MIM) and the matrix, while TOMM70 prefers substrates that are α-helical proteins destined for the outer or mitochondrial inner membrane.^4–6^

Though both receptors dynamically associate with the TOMM22/TOMM40 subcomplex, TOMM70 is considered to be more loosely associated with the core complex than TOMM20.^7–9^ TOMM70 typically migrates as a homodimer in Blue Native PAGE, which indicates its less stable interaction with the core complex.^10,11^ Conversely, when solubilized with mild detergents, TOMM20 also exhibits a loose association with the core complex but it migrates as a high-molecular-weight complex above 400 kDa in Blue Native PAGE, which is distinct from TOMM70.^12–14^ The delivery of substrate proteins to the receptors involves chaperones that prevent the aggregation of translated precursor proteins and target them to the corresponding receptors.^15^

In line with the proposed role of chaperones, *in vitro* import assays indicate that mitochondrial protein import occurs post-translationally. However, there is also increasing evidence that supports the idea of local translation of mRNAs at the mitochondrial surface and cotranslational import of mitochondrial proteins.^16–18^ This evidence includes the presence of ribosomes at the mitochondrial outer membrane (MOM),^19^ cofractionation of thousands of mRNAs with yeast mitochondria,^20^ or the identification of mRNAs by Ribo-Seq from ribosomes that were labeled by a biotin ligase targeted to the MOM.^21^ The translation-dependent accumulation of mRNAs at the MOM has also been reported in mammalian HEK293 cells.^16^ Proximity-labeling based RNA sequencing identified hundreds of mRNAs at the MOM, most of which encode mitochondrial proteins (mitoRNAs). Treatment with the translation inhibitor puromycin causes the detachment of most of these mRNAs from ribosomes, suggesting that their localization depends on translation, most likely via the mitochondrial targeting signal (MTS) in the nascent chain complex.^22,23^ Some of the TOM complex subunits may participate in cotranslational import as previous studies have shown that yeast TOM20 contributes to the cotranslational import of the mitochondrial proteins.^18^ In parallel, TOM70 may also contribute to some level to localized translation at the MOM to some extent, as its depletion in yeast and mammalian cells has been shown to reduce the levels of mitochondrial-localized mRNAs,^18,24^ or dissociates ribosomes associated with a subset of mitoRNAs.^17^

Furthermore, RNA-binding proteins (RBPs) that bind to mRNA encoding mitochondrial proteins can control the stability, translation, or localization of these mRNAs. Such proteins have been identified in yeast, *Drosophila*, and mammalian cells.^22^ In budding yeast, loss of the RBP Puf3p results in the delocalization of mRNAs to the MOM.^18,25^ This protein is involved not only in the localization of mitoRNAs to the MOM, but also in blocking the translation of these mRNAs while they are in transit. In mammals, two RBPs, Clustered Mitochondria Protein Homologue (CLUH) and Synaptojanin 2 Binding Protein (SYNJ2BP), have been extensively studied.^26^ CLUH preferentially binds mRNAs of nuclear-encoded mitochondrial proteins and its depletion results in reduced translation of the encoded proteins, leading to defects in mitochondrial morphology.^26^ SYNJ2BP has been identified as a component of RNA-protein complexes at the MOM. It is essential for the localization of its target mRNAs^27^ and the piggy-back travel of *PINK1* mRNA with mitochondria in neurons.^28^ SYNJ2BP knockdown redistributes *PINK1* mRNA into RNA granules and inhibits local mitophagy.^28^ SYNJ2BP specifically anchors its target mRNAs at the MOM under translation stress, facilitating their local translation and further import into mitochondria. Furthermore, loss of SYNJ2BP in HEK293 cells compromises the function of the OXPHOS (oxidative phosphorylation) complex.^27^

To compare the local interactomes of TOMM20 and TOMM70 with a focus on RBPs that are associated with the two receptors, we applied an APEX2-based proximity labeling approach. We fused the APEX2 enzyme to either TOMM20 or TOMM70 in HeLa cells. This revealed distinct sets of associated proteins, showing that each receptor subunit interacts with its own unique set of proteins, despite a large overlap. These proteins include translation factors, ribosomal proteins, and RBPs, such as SYNJ2BP, which specifically associates with TOMM20. This indicates an association of the translation machinery with the TOMM20 receptor subunit and suggests that it is more engaged than TOMM70 in localized translation at the mitochondrial outer membrane (MOM).

## Material and Methods

### Cell Culture

Each APEX2 fusion protein expressing cell line was generated by using a low passage HeLa 11ht parental cell line obtained from Dr. Kai Schönig (Zentralinstitut für Seelische Gesundheit Mannheim, Germany).^29^ Cells were always cultured in DMEM (Sigma) mixed with 10% fetal bovine serum (FBS), 110 mg mL^–1^ Sodium Pyruvate, and 1× Penicillin/Streptomycin at 37 °C with 5% CO_2_ and maintained by 200 μg mL^–1^ Hygromycin B (Sigma) and 200 μg mL^–1^ G418 (Sigma) as described before.^30^

### Plasmid Construction

All plasmids were generated via Gibson assembly. As a backbone for all constructs, plasmid RJP2501 (a pSF3 backbone vector containing GFP) was digested with restriction endonucleases *Bgl*II and *Pac*I to excise the GFP gene. The resulting linearized vector was used as a template to generate all plasmids via Gibson assembly (Supporting Table S1). Each corresponding fragment of the inset contained overlapping ends by the respective primers to facilitate the seamless joining of adjacent fragments via Gibson assembly reaction.

### Generation of Stable Cell Lines

To enable tunable doxycycline (DOX) induced expression and preserve isogenicity, the fusion protein expression cassette in the generated pSF3 plasmid for each cell line was stably integrated in the genome of the HeLa 11ht cells via recombinase-mediated cassette exchange (RMCE) as described before.^30^ Each doxycycline inducible cell line expressing APEX2 containing fusion protein was obtained by selection with 50 μM ganciclovir (Sigma).

### Immunofluorescence Microscopy

Around 25,000 cells were seeded on the coverslips coated with 0.2% Gelatin placed in 12-well plates. Cells were incubated either with or without 500 ng mL^–1^ doxycycline for 24 h. The samples were then thoroughly washed three times with PBS to remove media, fixed and permeabilized with ice-cold methanol for 10 min at −20 °C. The samples were washed two times with PBS containing 5 mM MgCl_2_ (PBSM) and once with 50 mM Glycine in PBSM. Cells were further incubated in 3% bovine serum albumin (BSA) diluted in PBSM (blocking solution) for 1 h. This was followed by incubation with the corresponding primary antibodies (mouse anti-V5 antibody, 1:1000 dilution; rabbit anti-TOM22 antibody, 1:200 dilution; mouse anti-TOM20 antibody, 1:200 dilution; rat anti-Flag antibody, 1:500 dilution; rabbit anti-TOM20 antibody, 1:200 dilution; further information on antibodies including provider can be found in Supporting Table S2) in the blocking solution for 1 h at room temperature or overnight at 4 °C. After washing three times with PBS, cells were incubated with Alexa Fluor coupled secondary antibodies (Invitrogen) at 1:1000 dilution in the same blocking buffer for 1 h. The cells were washed two times with PBS first and then incubated for 5 min with DAPI (0.1 μg mL^–1^) diluted in PBS. Cells were thoroughly washed again with PBS and mounted with ProLong Glass Antifade Mountant (Thermo). After overnight incubation at room temperature, the slides were stored at 4 °C until imaging. To analyze biotinylated proteins, biotinylation was performed directly on the cells seeded on the coverslips as explained below in the proximity labeling section. The cells were treated with the corresponding primary antibodies diluted in the blocking buffer as explained above. A Neutravidin conjugate labeled with Alexa Fluor 647 (prepared as per Invitrogen’s instructions) was mixed at 1:1000 dilution in the blocking buffer together with other corresponding secondary antibodies as mentioned above. After 1 h incubation at room temperature, the samples were washed thoroughly before mounting. All images were captured using a Zeiss LSM980 laser scanning confocal microscope (Carl Zeiss, Germany) in Airyscan imaging mode using 405, 488, 561, or 639 nm diode lasers, equipped with an Airyscan 2 detector and with a 60x oil objective. Captured images were first processed with Zeiss Zen software, and then with ImageJ/Fiji.^31^ For Figure 5D, line intensity measurements of TOMM20 and SYNJ2BP were performed in ImageJ/Fiji using the Plot Profile tool. Straight line ROIs of defined width were drawn across the structures of interest. The line intensity measurements (arbitrary units) of TOMM20-AF488 and SYNJ2BP-AF555 were quantified by measuring the mean intensity of corresponding channel fluorescence per area (square micrometers), background subtracted and normalized with values ranging from 0 (lowest) to 1 (highest).

### Proximity Labeling

For APEX2 labeling, cells were induced with doxycycline (DOX) or mock treated as described above. After 24 h, cells at around 85% confluency were incubated with fresh DMEM containing 0.5 mM biotin-phenol (BP; Iris Biotech GMBH) for 30 min at 37 °C. Biotinylation was initiated by adding H_2_O_2_ for 1 min at room temperature to the final concentration of 1 mM under constant agitation for each cell line.^32^ Control samples omitted either BP or H_2_O_2_ or uninduced cells were included for each APEX2-fusion protein expressing cell lines. Labeling reactions were quenched by removing media and immediate washing of cells with an equal volume of quenching DPBS solution that was prepared freshly after mixing DPBS with 10 mM sodium azide, 10 mM sodium ascorbate, and 5 mM Trolox. Cells were further washed two times with quenching DBPS solution, and once with PBS before trypsinization, and pelleting at 500*g*. The pellet was washed with PBS and immediately lysed in RIPA buffer (50 mM Tris, pH 7.5, 150 mM NaCl, 0.1% SDS, 1% Triton X-100, 0.5% sodium deoxycholate, and 1× cOmpleteprotease inhibitor cocktail) supplemented with 10 mM sodium azide, 10 mM sodium ascorbate, and 5 mM Trolox. The lysates were incubated on ice for 15 min, sonicated briefly, then centrifuged at 15,000*g* for 10 min at 4̊C. Protein concentration of the clarified lysates was determined with a Pierce 660 nm protein assay (Thermo, catalog no. 22660). 20 μg of whole cell lysate was combined with Laemmli buffer and boiled for 10 min. Lysates were separated on a 9% SDS-PAGE gel and transferred to nitrocellulose membrane. Membrane was stained by Ponceau S (0.1% w/v Ponceau S in 5% acetic acid (v/v)), afterward incubated in 3% BSA in TBST (0.1% Tween-20 in Tris-buffered saline) overnight. On the next day, the blot was incubated with 0.3 μg mL^–1^ streptavidin-HRP (Thermo) in TBS-T for 1 h at room temperature and further developed using the Pierce ECL Western Blotting-Substrate (Thermo).

### Seahorse Based Respiratory Analysis

Using a Seahorse XF96 Extracellular Flux Analyzer, a mitochondrial stress test was performed to measure the oxygen consumption rate (OCR) and extracellular acidification rate (ECAR) in HeLa cells. The cells were plated 24 h prior to the measurement. Following the manufacturer’s instructions, a sequential treatment with 4 μM Oligomycin (#S1478, Selleckchem), 5.6 μM CCCP (#C2759, Merck) and 4 μM Antimycin A (#A8674-50MG Merck)/0.8 μM Rotenone was done and the oxygen consumption was measured. After the respiratory analysis, the cells were fixed with 4% paraformaldehyde containing Hoechst stain at 1:10000 for 5 min and then washed with PBS. Hoechst was used to stain the nucleus, and the intensity was measured using a SpectraMax M2e plate reader. The nuclear stain intensity was used to normalize the values obtained from the respiratory analysis to account for cell number in each well.

### Cell Fractionation

For each cell fractionation experiment, cells were induced with 500 ng mL^–1^ doxycycline for 24 h or mock treated. Following induction, the media was discarded from the around 85% confluent cell culture dishes, and cells were washed with PBS twice and then collected in 15 mL centrifuge tubes. Cells were pelleted at 500*g*, 3 min at 4 °C, the supernatant discarded, and the cell pellet resuspended in HMS-A buffer^33^ (0.22 M mannitol, 0.02 M HEPES-KOH, pH 7.6, 1 mM EDTA, 0.075 M sucrose, 0.1% BSA, 1× cOmpleteprotease inhibitor cocktail (Roche)). The suspension was incubated for 10 min on ice, transferred to a homogenizer (Sigma) and lysed with 30 cycles on ice. The lysates were centrifuged at 900*g*, 5 min at 4 °C. The supernatant was collected and centrifuged again for 9000*g*, 15 min at 4 °C, resulting in a crude mitochondrial fraction in the pellet and a cytosolic fraction in the supernatant. The mitochondrial pellet was further washed with HMS-B buffer (HMS-A buffer without BSA) and mitochondria pelleted again at 10,000*g*, 10 min, 4 °C. To solubilize mitochondria, the pellet was resuspended again in HMS-B buffer with digitonin (4:1 (w/w) ratio of detergent to the protein) and incubated for 30 min at 4 °C. Nonsolubilized material was removed via centrifugation (13,000*g*, 10 min, 4 °C). The solubilized mitochondrial fraction was collected and denatured for Western blot. Around 100 μg solubilized mitochondrial fraction was separated either on a 12% SDS-PAGE gel (for TOMM20-APEX2 detection) or a 10% SDS-PAGE gel (for TOMM70-APEX2 detection). For expression validation in Figure 1F, blots were incubated with primary antibodies 1 h at room temperature or overnight at 4 °C. Primary antibodies include mouse anti-TOM20 (1:250 dilution), rabbit anti-TOM40 (1:500 dilution); mouse anti-TOM70 (1:500 dilution), and mouse anti-GAPDH (1:1000 dilution). Further information on antibodies including provider can be found on Supporting Table S2.

**Figure 1:**
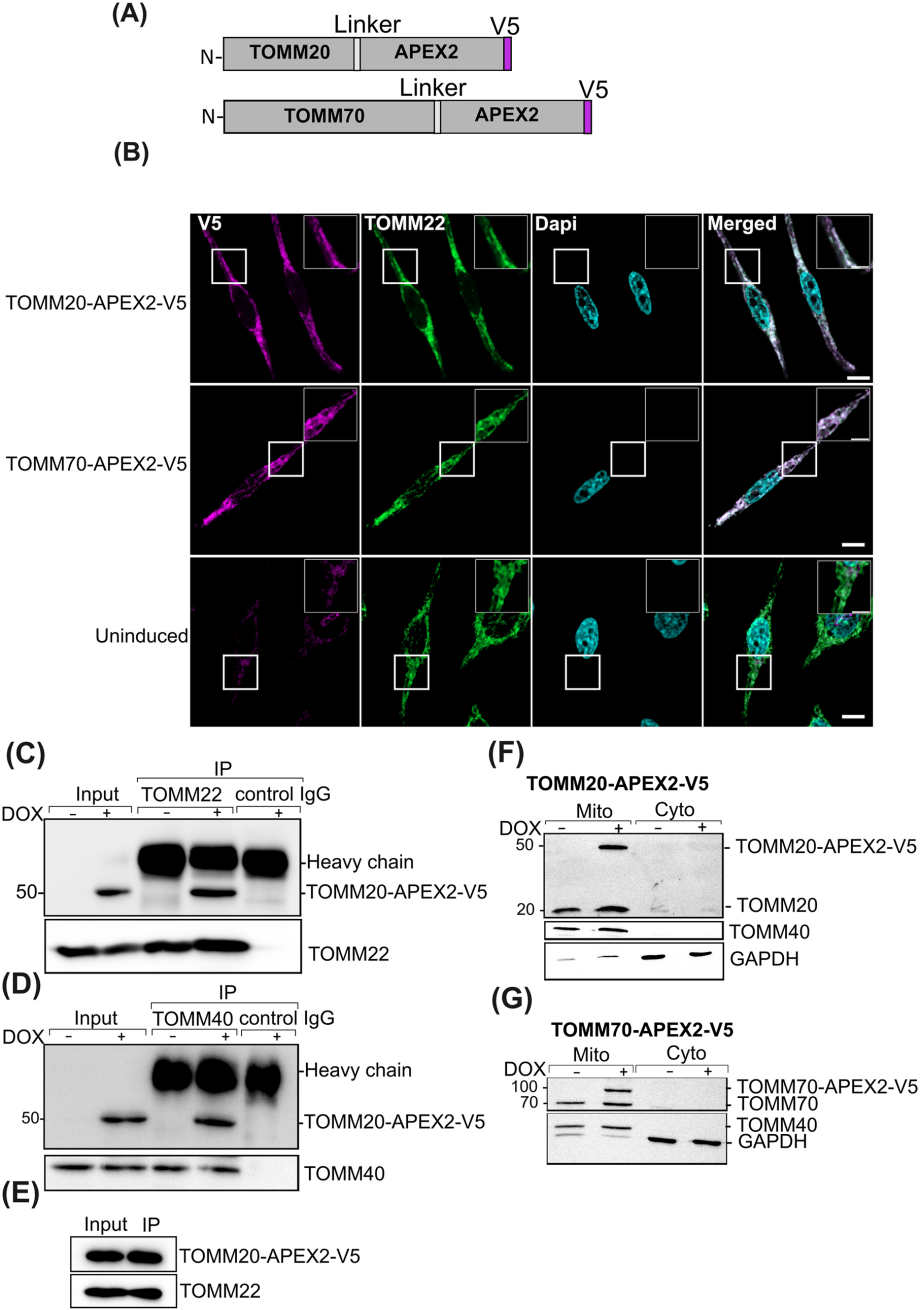
Functional expression of TOMM20- and TOMM70-APEX2 fusion proteins. (A) Sketches of TOMM20-APEX2 and TOMM70-APEX2 fusion proteins. APEX2 and V5 tags are fused to the C-terminus of the target protein via a short intervening linker sequence of 10 amino acids. (B) Confocal fluorescence imaging of APEX2 fusion proteins in HeLa cells. Fusion protein localization is revealed after 24 h induction with doxycycline (DOX) by immunolabeling with an antibody directed against the V5 tag (Magenta). Endogenous TOM complex is visualized using an anti-TOM22 antibody (Green). Nuclei are stained with DAPI (cyan). Scale bar: 10 μm. Insets in the uppermost right of each panel are magnifications of the boxed areas (Scale bar: 5 μm). (C, D) Western blot showing interaction of TOMM20-APEX2 fusion protein with endogenous TOM complex subunits after pull-down. Mitochondrial TOM complexes of cells expressing TOMM20-APEX2 (DOX+) or of uninduced cells (DOX−) were specifically immunoprecipitated using either anti-TOMM22 (C), or anti-TOMM40 antibodies (D). Input and immunoprecipitates were probed for the presence of TOMM20-APEX2 (using anti-V5 antibody), and with anti-TOMM22 or anti-TOMM40 antibodies. Numbers on the left of each panel refer to protein mass in kDa. (E) Western blot showing the coimmunoprecipation of endogenous TOMM22 with TOMM20-APEX2 using anti-V5 beads that capture the fusion protein. (F) Subcellular fractionation demonstrating enrichment of TOMM20-APEX2 in a mitochondrial (mito) versus cytoplasmic (cyto) fraction without (−) or with (+) the induction of fusion protein expression with DOX. Isolated fractions were analyzed with anti-TOMM40 and anti-TOMM20, which detected endogenous TOMM20 (∼20 kDa) and induced TOMM20-APEX2 (∼50 kDa). GAPDH is used as a cytosolic marker protein. (G) Western blot analysis of mitochondrial and cytosolic fractions of TOMM70-APEX2 expressing cells. Endogenous (70 kDa) and APEX2 fused TOMM70 (∼100 kDa) proteins are detected by an anti-TOMM70 antibody. Cytosolic fraction is validated by presence of GAPDH.

### Co-Immunoprecipitation

Mitochondria were isolated and solubilized as described above. Around 250 μg of solubilized mitochondria were used for each copurification and incubated with 10 μg of anti-TOM22 (Abcam) or 20 μg of anti-TOM40 (Proteintech) antibodies for 2 h at 4 °C. Normal rabbit IgG was used as an isotype control. After incubation, prewashed protein A agarose beads were added and incubated for another hour at room temperature. Beads were washed three times with HMS-B buffer and proteins eluted with 2 × Laemmli buffer at 95 °C for 10 min. The eluate was collected, separated on a 12% SDS-PAGE gel before detection of proteins by Western blotting. The primary antibodies used were rabbit anti-TOM22 (1:500 dilution), rabbit anti-tomm40 (1:500 dilution), and mouse anti-V5 (1:1000 dilution). For V5 coimmunoprecipitation, V5 beads (ChromoTek) were prewashed with HMS-B buffer three times. Solubilized mitochondria were cross-linked first with 1 mM DSP for 1 h at 4 °C followed by quenching using 1 M Tris-HCl, pH 7.5 to a final concentration of 50 mM for 15 min. The cross-linked solubilized mitochondria were purified through a filter column and incubated with 50 μL of V5 beads in HMS-B for 1 h at room temperature. Beads were washed three times with HMS-B buffer, eluted in 2× Laemmli buffer with 0.1 M DTT at 95 °C for 10 min, and further analyzed by Western blot. After boiling the beads in the Laemmli buffer, the eluate was collected, separated on a 12% SDS-PAGE gel before detection of proteins by Western blotting. The primary antibodies used were rabbit anti-APEX2 (1:3000 dilution), rabbit anti-TOM22 (1:500 dilution), and rabbit anti-SYNJ2BP (1:500 dilution). Further information on antibodies including provider can be found on Supporting Table S2.

### Streptavidin Pulldown for Mass Spectrometry (MS) Sample Preparation

Around 1.5 million cells for each replicate were induced with DOX for 24 h. Controls for uninduced cells and puromycin treatment were seeded at the same time. Next day, cells having 85% confluency were biotinylated and further lysed in the RIPA buffer as described above. For puromycin treatment, cells were treated with 200 μM puromycin and 0.5 mM BP for 30 min at 37 °C.^16,27^ APEX2 labeling was induced by H_2_O_2_ treatment for 1 min as described above. 50 μL streptavidin-coated magnetic beads (Pierce, catalog no. 22660) were used for each replicate and washed three times with RIPA buffer with end-to-end rotation. Prewashed beads were incubated with 120 μg of lysate for each replicate for 2 h at room temperature under constant end-to-end rotation. For capturing of biotinylated proteins, we followed a published protocol.^32^ After capturing, beads were washed twice with 1 mL of RIPA lysis buffer for 2 min, once with 1 M KCl for 2 min, once with 0.1 M Na_2_CO_3_ for around 10 s, once with 2 M urea in 10 mM Tris-HCl (pH 8.0) for around 10 s, and again twice with 1 mL RIPA lysis buffer for 2 min at room temperature. The beads were subsequently transferred to fresh tubes and washed briefly first with RIPA buffer, once with 1 mL wash buffer (75 mM NaCl in 50 mM Tris-HCL pH 7.5) and then processed for LC-MS/MS analysis as follows.

### Sample Preparation for MS

On bead captured interaction partners of TOMM20, TOMM70, or otherwise tagged by NES-, and Mito-APEX2 markers were resuspended in 30 μL of denaturation buffer (6 M urea, 2 M thiourea, 10 mM Tris, pH 8). Disulfide bonds were reduced with 10 mM of DTT for 1 h and further alkylated with 55 mM of iodoacetamide for 1h in the dark. Proteins were predigested with 2 μL of LysC (Lysyl endopeptidase; Wako Chemicals) for 3 h. For overnight digestion with 2 μL of trypsin (Promega Corporation) samples were diluted 4-fold with 50 mM ammonium bicarbonate. On the next day, beads were pelleted, the supernatant was transferred to a new tube and the digestion was stopped by adding 1% of TFA. Contaminants were removed by the PreOmics’ Phoenix kit prior to their submission to the MS.

### LC-MS Measurement and Data Analysis

All proximity labeled samples were analyzed on a Q Exactive HF mass spectrometer (Thermo Fisher Scientific) online-coupled to an Easy-nLC 1200 UHPLC (Thermo Fisher Scientific). Prior to MS-based analysis peptides were separated on an in-house packed (ReproSil-Pur C18-AQ 1.9 μm resin [Dr Maisch GmbH Ltd.]) 20 cm analytical column (75 μm ID PicoTip fused silica emitter [New Objective]). For gradient elution of the peptides solvent A (0.1% FA) and solvent B (0.1% FA in 80% ACN) were used across a 60 min gradient with a flow rate of 200 nL/min at 40 °C. The mass spectrometer was operated in positive ion and in data-dependent acquisition mode. MS1 spectra were acquired over a scan range from 300 to 1650 *m*/*z* at resolution 60k. The top seven most abundant peptides were selected for isolation within an isolation window of 1.4 *m*/*z*, HCD fragmentation with nce set to 27 and a maximum injection time of 110 ms/AGC target 1e5. MS2 spectra were acquired at resolution 60k. Peptides were excluded from reanalyzing for 30 s.

Generated raw files were further processed with the MaxQuant software suite,^34^ version 2.2.0.0. All parameters were kept as default with trypsin specific digestion mode selected and two missed cleavages allowed. As a quantification method, label-free quantification was selected with minimal ratio count of 1. Cysteine carbamidomethylation was selected as fixed as well as methionine oxidation and protein N-terminal acetylation as variable modifications. Match between runs was allowed across all raw files. All spectra were searched against the UniProt *Homo sapiens* database (downloaded on December 16, 2022, 103 830 entries) and commonly observed contaminants.

For downstream data analysis, only protein groups that were not identified as contaminants or by site were considered. Data were analyzed using Perseus (version 1.6.7.0) with annotation for cellular compartment and biological function based on Gene Ontology and MitoCarta3.0.^35^ Label-free quantification (LFQ) intensities were log_10_-transformed prior to statistical analysis. Within biological replicates, proteins were required to be quantified in at least two of three replicates (or in ≥ 75% of replicates where more than three were available). Missing values were imputed in Perseus using the default method of random number generation from a normal distribution, assuming a Gaussian distribution with a width of 0.3 and a downshift of 1.9, applied separately to each sample.^36^ Differential protein abundance was assessed using a two-sample Student’s *t* test in Perseus with parameters S0 = 0 and side = both, i.e., without SAM-based hyperbolic thresholding. In this analysis, the Perseus “Difference” column corresponds to the log_10_ fold-change between group means. Proteins were classified as significantly up- or downregulated when they fulfilled both criteria: (i) a *t* test *p*-value <0.05 and (ii) a fold-change equivalent to a log_2_ difference of at least 3. To implement this combined cutoff, proteins were required to have an observed −log_10_(p) greater than the calculated hyperbolic threshold line, ensuring that both statistical significance and effect size were considered. Unless otherwise stated, the *p*-values reported are raw values from the *t* test and were not adjusted for multiple testing. Additional data visualization was performed within the R environment (box plots, venn diagrams) or with alternative Shiny applications such as UpSet plots.^37^ Network analyses were performed in the STRING database with annotations derived from the STRING server itself.^38^ The mass spectrometry proteomics data have been deposited in the ProteomeXchange Consortium via the PRIDE partner repository^39^ with the data set identifier PXD057097 (doi:10.6019/PXD057097).

### AlphaFold Based Prediction of Protein Interaction

AlphaFold3 server^40^ was used to predict the structure of protein complexes of TOMM20 (uniport Q15388, aa 25–145) or TOMM70 (O94826, aa 60–608) with SYNJ2BP (P57105, aa 1–117). The top-ranked model obtained from AlphaFold 3 was chosen as the baseline structure for interface analysis with ChimeraX^41^ (version 1.10.1). Residues at the interface were defined as those that have at least one heavy atom that is less than 8 Å away from any heavy atom of the opposing peptide chain. Interactions were further filtered using the Predicted Aligned Error (PAE), retaining only residue pairs with a maximum PAE ≤ 5 Å to ensure high-confidence contacts.

## Results

### Functional TOMM20-APEX2 and TOMM70-APEX2 Fusion Proteins Are Expressed in the HeLa Cells

To characterize the interactomes of the two main receptor subunits of the human TOM complex, we generated expression constructs targeted for integration into the HeLa-EM2–11ht genome (see Methods). In both hybrid proteins, the APEX2 proximity labeling enzyme^42^ is located at the cytoplasmic carboxyl (C−) terminus of the respective TOMM protein, separated by a short linker sequence (GGSGDPPVAT). Both fusion proteins contain an additional V5 epitope tag^43^ for detection (Figure 1A) and are expressed from an inducible pTet promoter by addition of doxycycline (DOX) to the medium.

We initially used the V5 tag to test if the fusion proteins are targeted to mitochondria (Figure 1B). After 24 h induction, immunofluorescence microscopy at super resolution revealed a colocalization of TOMM20-APEX2-V5 or TOMM70-APEX2-V5 with a mitochondrial marker (TOMM22), which indicates the correct targeting of the fusion proteins. Furthermore, mitochondrial morphology remained unchanged, which suggested that the fusion proteins have no negative impact on mitochondrial function.

To test for correct association of TOMM20-APEX2 with the TOM complex we performed pull down assays with endogenous TOM complex subunits. TOMM20-APEX2 expression was induced for 24 h prior to lysis, mitochondria were isolated and solubilized with a digitonin-containing buffer. Detergent extracts were subjected to coimmunoprecipitation using an anti-TOMM22 or anti-TOMM40 antibody and the fusion proteins detected via the V5 tag. The 49 kDa TOMM20-APEX2 protein fusion was found in both immunoprecipitates (Figure 1C,D). In a reciprocal coimmunoprecipitation experiment, using anti-V5 beads we copurified TOMM22, as shown by Western blotting using an anti-TOMM22 antibody (Figure 1E). These experiments confirmed that TOMM20-APEX2 associates with both members of the endogenous TOM complex. We also analyzed integration of both fusion proteins into mitochondria by subcellular fractionation. Like endogenous TOMM40 and TOMM20, TOMM20-APEX2 (detected by an anti-TOMM20 antibody) is highly enriched in the mitochondrial fraction, whereas glyceraldehyde-3-phosphate dehydrogenase (GAPDH) is primarily detected in the cytoplasmic fraction (Figure 1F). Subcellular cofractionation was also used to assess distribution of TOMM70-APEX2. Detection of the fusion protein and the endogenous TOMM70 via an anti-TOMM70 antibody reveals similar distribution patterns in mitochondrial versus cytosolic fractions, indicative of correct targeting of the fusion protein to mitochondria (Figure 1G). Importantly, these experiments also revealed similar expression levels of endogenous TOMM20 or TOMM70 and the corresponding fusion proteins TOMM20-APEX or TOMM70-APEX, respectively.

Finally, we performed mitochondrial respiration assays to test if expression of the APEX2-fused variants of TOMM20 and TOMM70 interfere with mitochondrial function (Supporting Figure S1). Upon DOX based induction of TOMM20-APEX2, no significant differences in several functional parameters including basal respiration or ATP production were detected between Hela cells with or without the integrated construct. Nonsignificant deviation in some of these parameters was seen in cells containing the TOMM70-APEX2 construct. Since this occurred independently of the induction of TOMM70-APEX2 by DOX, we conclude that neither fusion protein disturbs mitochondrial function. Together with the aforementioned observed interaction (Figure 1) we thus conclude that both fusion proteins are functional.

### TOMM20-APEX2 and TOMM70-APEX2 Fusion Proteins Show Specific and Localized Biotinylation Activity

Since correct localization of the TOMM20- and TOMM70-APEX2 fusion proteins to mitochondria (Figure 1) as well as their copurification with TOMM22 and TOMM40 proteins suggest that both APEX2 fusions are correctly integrated into the TOM complex, we next tested for biotinylation activity of the fusion proteins. Besides endogenous biotinylated proteins that can be detected under all conditions, additional biotinylation in cells with integrated TOMM20- or TOMM70-APEX2 constructs could only be detected upon expression of the respective protein (Supporting Figure S2, “DOX”), addition of biotin-phenol (“BP”), and of H_2_O_2_ (“H_2_O_2_”). We also verified biotinylation by *in situ* labeling of biotinylated proteins, using neutravidin-Alexa647 (Figure 2).

**Figure 2:**
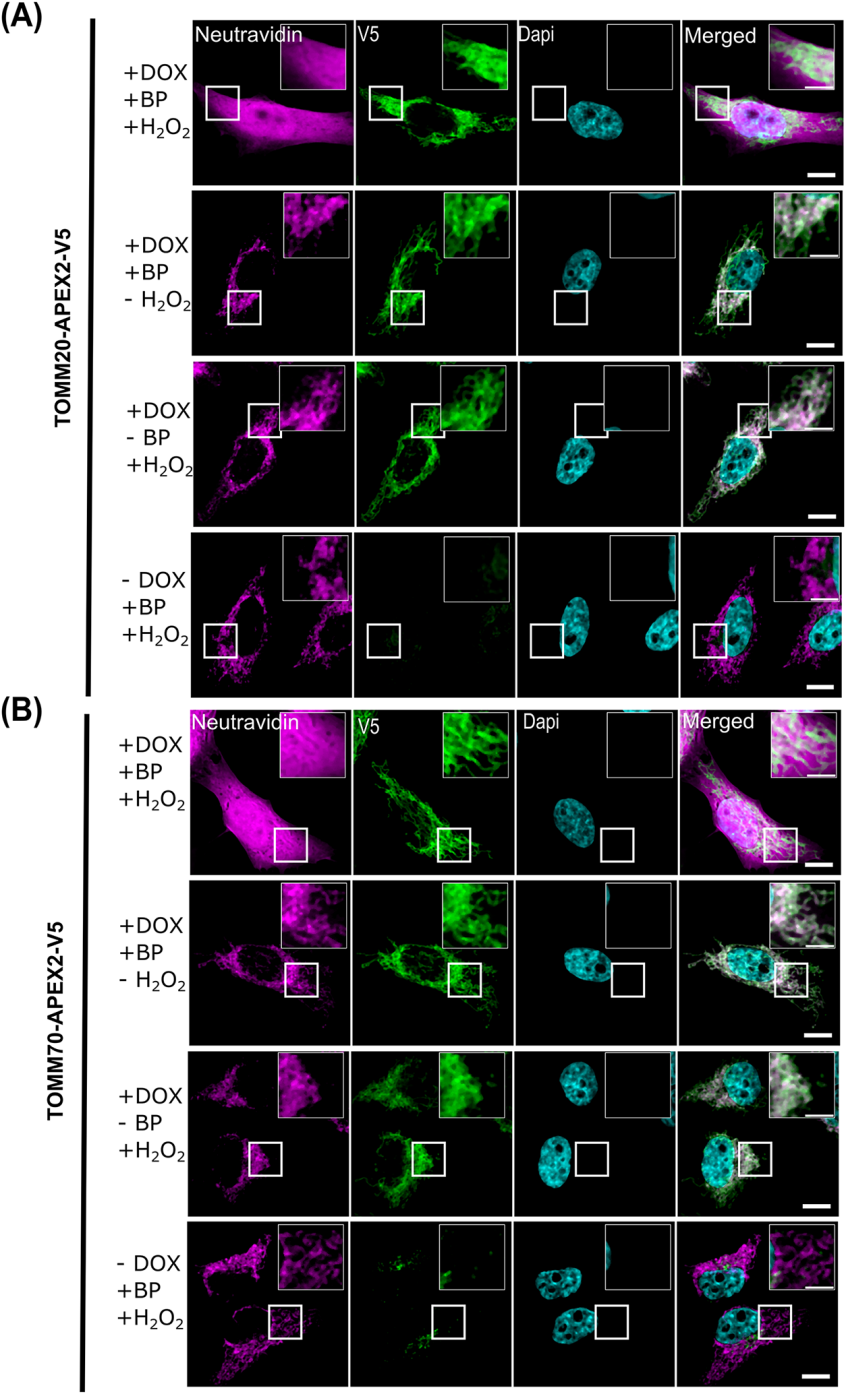
Local biotinylation at mitochondria by TOMM20- and TOMM70-APEX2. Confocal fluorescence imaging of APEX2-mediated biotinylation in HeLa cells stably expressing the indicated APEX2 fusion proteins. (A) TOMM20-APEX2-V5 (B) TOMM70-APEX2-V5. After 24 h of doxycycline (DOX) induction, biotinylation was triggered for 1 min with biotin-phenol (BP) and H_2_O_2_ and cells were subsequently fixed. Magenta channel indicates biotinylation as detected by neutravidin-coupled Alexa 647 and green channel indicates detection of the APEX2 fusion protein via the V5 tag. Endogenous mitochondrial biotinylated proteins are visible if either H_2_O_2_ or BP was omitted, or when the cells were not induced. Nuclei are stained with DAPI (cyan). Insets in the uppermost right of each panel are magnifications of the boxed areas (Scale bar: 10 μm; scale bar in inset: 5 μm).

Even under conditions that do not allow APEX activity (e.g., no expression, no BP, no H_2_O_2_), a neutravidin-Alexa647 signal is detectable that overlaps with the mitochondrial location of the APEX2 fusion proteins. This most likely reflects detection of endogenous biotinylated proteins such as mitochondrial pyruvate carboxylase or acetyl-CoA-carboxylase.^44^ Only HeLa cells expressing the TOMM20- and TOMM70-APEX2 fusion proteins show an additional, and stronger biotin-dependent fluorescence (Figure 2A,B, left column), indicating an active APEX2 enzyme. Surprisingly, the biotinylation seen in TOMM20-APEX and TOMM70-APEX expressing cells was not restricted to the mitochondrial location. However, this has been seen before for TOMM20-APEX2 ^45^ and has been explained to depend on the cytosolic orientation of the APEX2 part of the fusion protein to label cytosolic components and the diffusion of the activated biotin-phenoxyl radicals.

Since the TOMM20- and TOMM70-APEX2 fusion proteins are expressed at endogenous levels and correctly associated with endogenous TOM complex proteins, we next aimed to identify their corresponding interactomes. To control for non-specific interactions, we chose two additional doxycycline-controlled APEX2 fusion proteins (Supporting Figure S3A).^32,42^ Mito-APEX2 targets the APEX2 enzyme to the mitochondrial matrix, whereas APEX2-NES targets APEX2 to the cytosol due to a carboxyterminal nuclear export signal (NES)^42^ (Supporting Figure S3B,C). Both control APEX2 proteins are functional and only increase detectable biotinylation in the presence of BP and H_2_O_2_ (Supporting Figure S4A). In case of Mito-APEX2, neutravidin-Alexa647 staining of biotinylated proteins *in situ* (Supporting Figure S4B) shows a very similar distribution to that of the enzyme itself, most likely due to the confinement of the activated phenoxyl-biotin radicals in the mitochondrial matrix. As expected, the distribution of proteins biotinylated by APEX2-NES is more diffuse and mimics the distribution of cytoplasmic proteins.

### TOMM20- and TOMM70-APEX2 Proximitomes Differ at the MOM-Cytoplasm Interface

Six independent replicates for TOMM20-APEX2, and three for TOMM70-APEX2, APEX2-NES, and Mito-APEX2 were analyzed by bottom-up proteomics. The usage of the six replicates in case of TOMM20-APEX2 boosted the number of identifications and made the data set more robust without introducing additional variance compared to three replicates (Supporting Figure S5). In addition, we included three replicates of control experiments performed with cells not expressing the corresponding TOM complex resident bait proteins (“-DOX”). Biotinylation with BP and H_2_O_2_, quenching, lysis and capturing was essentially done according to a previously published protocol^32^ (see Methods). The captured proteins were further measured by liquid chromatography-tandem mass spectrometry (LC-MS) after on-bead tryptic digestion. Downstream data processing was performed as label free quantification (LFQ), and imputation of missing values was applied during subsequent data analysis to increase the number of identifications (see Methods). With this approach we identified in total 2177 protein groups of which 499 are annotated for mitochondrial localization by MitoCarta3.0.^35^ Except for -DOX controls, 1300 to 1700 protein groups were identified with the highest number of mitochondrial localized proteins for Mito-APEX2 (Supporting Figure S6A). Overall, we observed an excellent correlation between the replicates (Supporting Table S3; Figure S6B). We next evaluated the suborganelle distribution of proteins biotinylated by TOMM20- and TOMM70-APEX2 (Supporting Figure S6C). We will use the term “proximitome” for these proteins to stress that these proteins are proximal to bait but might not directly interact. Around 1700 proteins were identified in TOMM20-APEX2 and TOMM70-APEX2 indicating a very similar number of biotinylated proteins. Besides the expected mitochondrial proteins, we also surprisingly observed enrichment of nuclear proteins, ribosomal, and cytosolic proteins in the proximitomes of both bait proteins. We currently have no explanation for the appearance of nuclear proteins but they have previously been observed in the interactome of a TOMM20-TurboID^46^ protein fusion. The cytoplasmic proteins observed in TOMM20- and TOMM70-APEX2 proximitomes might stem from the relative positioning of the APEX2 in the fusion protein. The APEX2 parts of both fusion proteins are facing toward the cytosol, hence we expected them to not only biotinylate MOM proteins or substrate proteins of the receptors but also, due to the release and diffusion of the phenoxyl-biotin radicals, proteins in the surrounding cytoplasm.^45^

To check that the TOMM20- and TOMM70-APEX2 bait constructs allow us to study their respective proximitomes, we compared the biotinylated proteomes generated by these two baits with those from cells not induced for expression of the corresponding APEX2 constructs (Figure 3A,B; Supporting Tables S4 and S5). As expected, fewer proteins were identified in the uninduced controls, making imputation of especially low abundant proteins for these samples mandatory (Supporting Figure S6D–G). Among the significantly enriched proteins (*p*-value ≤ 0.05) we identified additional subunits of the TOM complex (e.g., TOMM40) or other MOM proteins (21 in case of TOMM20-APEX2, seven in case of TOMM70-APEX2), which demonstrates the effectiveness of our approach. Surprisingly, whereas TOMM70 was present in the data set of proteins proximal to TOMM20-APEX2 and identified as a biotinylated protein in TOMM70-APEX2 expressing cells, TOMM20 was not. A closer look at the raw data revealed that it had been identified only by one peptide, and due to the raw data processing settings, it was not considered for further analysis. The low number of TOMM20 peptides may be due to the small size of the 20-kDa protein itself, which generates few peptides, and the limited accessibility of the small, cytosol-facing domain of TOMM20 ^47^ to activated biotin. In addition, the cytoplasmic domain contains few aromatic amino acids available for reaction with the biotin-phenoxyl radical generated by APEX2.^32,48^

**Figure 3:**
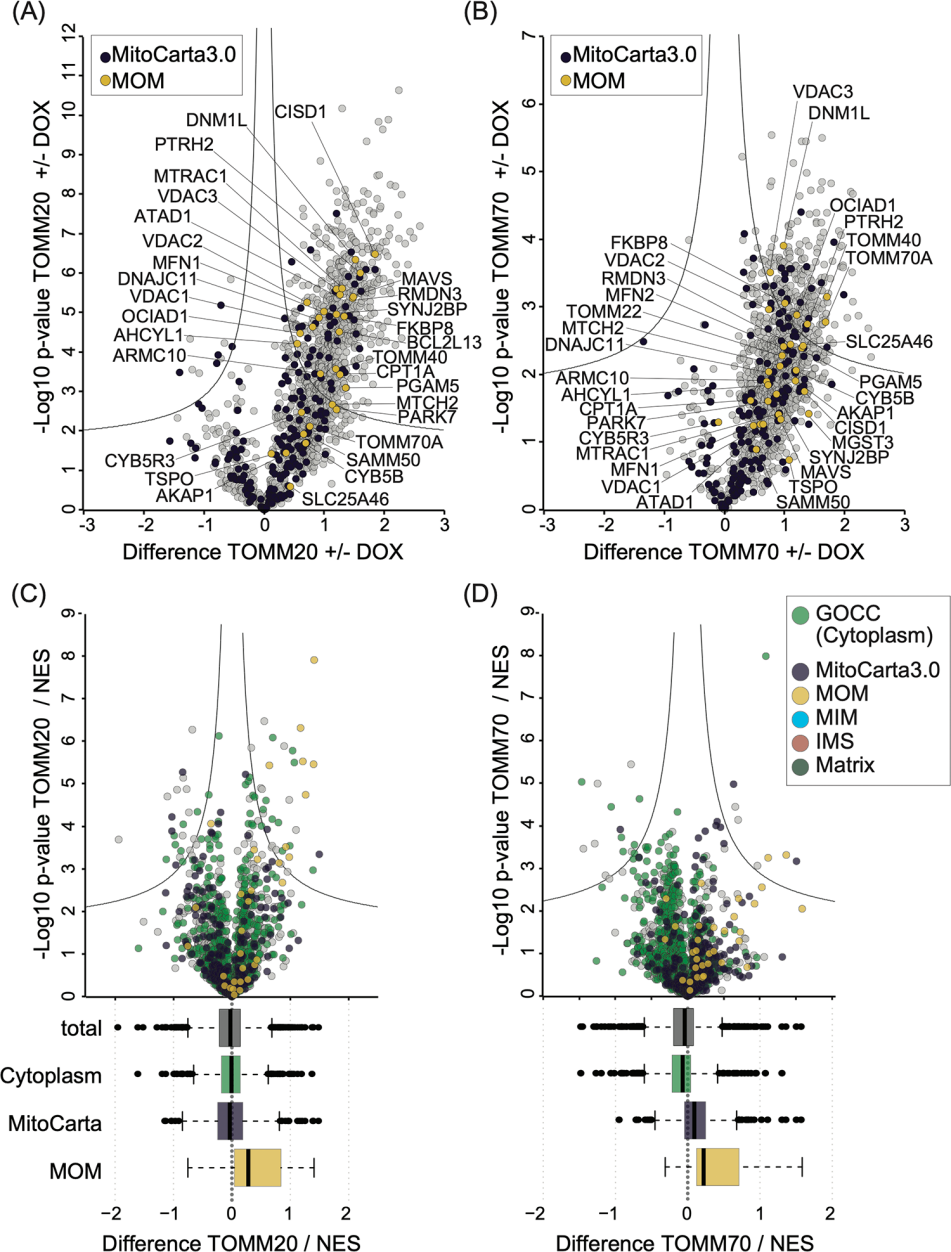
Proximitomes of TOMM20-APEX2 and TOMM70-APEX2 at the mitochondrial outer membrane. Proximitome of TOMM20-APEX2 (A) and TOMM70-APEX2 (B) including many mitochondrial proteins compared to their respective uninduced (-DOX) controls. Volcano plots for TOMM20-APEX2 (C) and TOMM70-APEX2 (D) against APEX2-NES proximitome. Highlighted are proteins annotated for cytoplasmic localization (based on GOCC) and mitochondrial localization (MitoCarta3.0). Boxplot shows the distribution of total proteins compared to proteins annotated for mitochondrial, and cytosolic localization for TOMM20- and TOMM70-APEX2 against APEX2-NES. Indicated are thresholds for significantly enriched proteins (*p*-value 5%, difference 3).

Next, we checked if the proximitomes of TOMM20- and TOMM70-APEX2 indeed reflect processes occurring at the MOM-Cytoplasm interface. We compared the pattern of biotinylated proteins with that of a cytosolically targeted APEX2 (APEX2-NES). Importantly, a first observation made when comparing the proximitome of TOMM20- and TOMM70-APEX2 with that of APEX2-NES was that MOM proteins were expectedly more enriched in the TOMM20- and TOMM70-APEX2 proximitomes (Figure 3C,D; Supporting Tables S6 and S7). However, several proteins annotated as cytoplasmic were also enriched in the TOMM20- or TOMM70-APEX2 proximitomes when compared to the APEX2-NES. As discussed before this has been already seen in related studies^45^ and might reflect biotinylation of proteins at the cytoplasmic face of mitochondria or by biotin-peroxyl radicals released into the cytoplasm.

Since one of our aims was to elucidate the extent of RBPs and factors involved in (local) translation that are associated with TOMM20 or TOMM70, we next analyzed our data set for the enrichment of proteins related to RNA function (GO:0003723). While no RBPs were identified as enriched in the TOMM70-APEX2 proximitome when compared to that of a cytoplasmic APEX2, seven RBPs (i.e., 23% of all enriched proteins compared the APEX2-NES) were found in the TOMM20-APEX2 proximitome, including SYNJ2BP, PAIP1, MAVS, and PABPC4L (Supporting Tables S6 and S7).

We also compared the proximitome of TOMM20- and TOMM70-APEX2 against that of a matrix-targeted APEX2 (Mito-APEX2; Supporting Figure S7; Tables S8 and S9). In comparison to Mito-APEX2 both MTS receptor APEX2 baits showed higher enrichment of annotated MOM proteins but little or no enrichment of matrix or MIM proteins. This indicates that our APEX2-based approach mainly targets proteins localized to the MOM or to the MOM-Cytoplasm interface in close proximity to the TOM complex (Supporting Figure S7) rather than substrate proteins bound to the receptors in transit. Compared to TOMM70-APEX2, for TOMM20-APEX2 we identified more MOM annotated proteins (14 as compared to six). TOMM20 appears to interact with more MOM proteins compared to TOMM70, probably due to a more stable association with the MOM or the TOM complex.^7–9^

### RNA-Binding Proteins and Translation Factors Are Part of the TOMM20-APEX2 Proximitome

After we validated the suitability of our experimental design to study the proximitome of TOMM20- and TOMM70-APEX2 at the interface of MOM and cytoplasm, we next directly compared the proximitomes of TOMM20- and TOMM70-APEX2 with each other (differential proximitome; Figure 4A).

**Figure 4:**
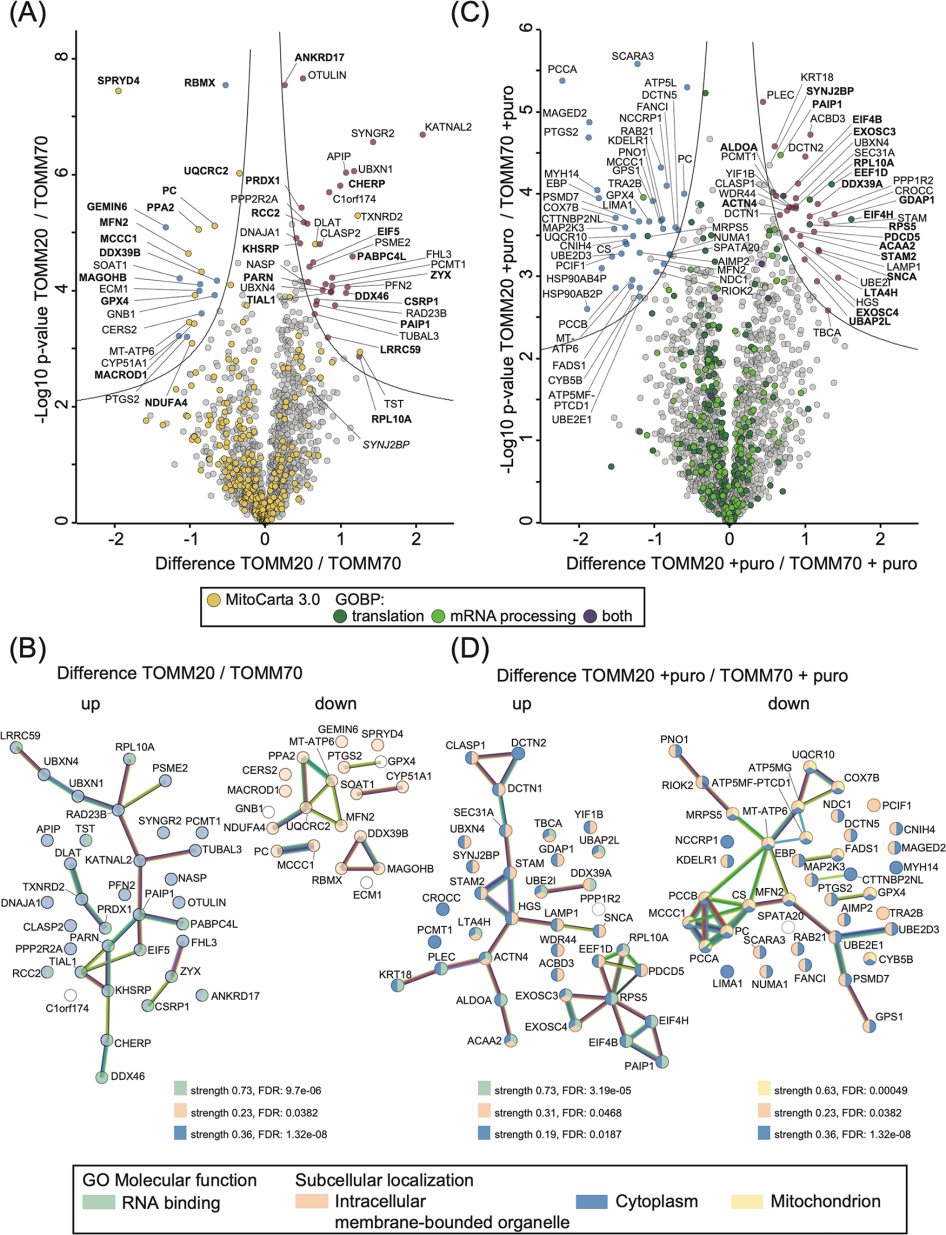
Proximitomes of TOMM20-APEX2 and TOMM70-APEX2 differ in their enrichment of RNA-binding proteins. (A) Volcano plot of a differential TOMM20-APEX2 versus TOMM70-APEX2 proximitome. Indicated as a line is the threshold for significantly enriched proteins (*p*-value 5%, difference 3). Yellow highlighted proteins are annotated for mitochondrial localization (MitoCarta3.0), proteins in bold typeface are annotated RNA-binding proteins. (B) STRING analysis of selected proteins from the differential proximitome of TOMM20-APEX2 vs TOMM70-APEX2 (A), showing connections between enriched candidates of TOMM20 (left, “up”), and enriched candidates of TOMM70 (right, “down”). Proteins with the annotated GO molecular function “RNA binding” (green) and subcellular localizations “cytoplasm” (blue), “organelle” (orange’), and mitochondrion’ (yellow) are shown. (C) Volcano plot of a differential TOMM20-APEX2 versus TOMM70-APEX2 proximitome after puromycin treatment (+puro). Proteins in bold typeface are annotated RNA-binding proteins. Highlighted are the proteins annotated for a role in translation and mRNA processing. (D) STRING analysis of selected proteins from the differential proximitome of TOMM20-APEX2 (+ puro) vs TOMM70-APEX2 (+puro) from volcano plot (C) showing enriched candidates of TOMM20 (left, “up”), and enriched candidates of TOMM70 (right, “down”). Proteins are highlighted according to GO molecular function “RNA binding” and subcellular localization. Strength and False discovery rate (FDR) of each pathway are indicated.

While most mitochondrial proteins are similarly enriched in the proximitomes of both baits, 20 proteins were identified to be significantly overrepresented in the TOMM70-APEX2 proximitome including three proteins annotated as exclusively mitochondrial; NDUFA4 (MIM), PPA2 (matrix), and seven proteins annotated as dual or multiple localized including pyruvate carboxylase (matrix, cytosol), MCCC1 (matrix, cytosol), UQCRC2 (MIM, nucleoplasm), MFN2 (MOM, ER, cytosol), GPX4 (MIM, extracellular space, nuclear envelope, cytosol), MACROD1 (matrix, cytoplasm, nucleus), and SRRYD4 (cytosol, matrix) (Supporting Table S10). A total of 35 proteins were found to be significantly overrepresented in the TOMM20-APEX2 proximitome, among them four proteins annotated as mitochondrial including DLAT (Matrix), TST (Matrix, extracellular matrix), TXNRD2 (Matrix, cytosol), and DNAJA1 (MOM, ER, cytosol, nucleus, extracellular matrix) (Figure 4A; Supporting Table S10). The identified MIM, IMS or matrix proteins might reflect precursors that were caught in transit while interacting with the TOMM proteins or be mature proteins that were labeled by biotin-phenoxyl radicals that traversed the MOM. However, since only very few MTS containing peptides were identified, distinguishing between precursors in transit and mature proteins at their destination site was not possible. We conclude from these experiments that APEX2 based labeling identifies more MOM proteins when the enzyme is fused to TOMM20 than TOMM70. In addition, we detect an enrichment of RNA-related proteins preferentially with TOMM20-APEX2.

Cytoplasmic RBPs including CHERP, PAIP1, KHSRP/FUBP2, PABPC4L, TIAL1, PARN, DDX46, ANKRD17, LRRC59, and components of the translational machinery like RPL10A and EIF5 were identified as significantly enriched in TOMM20-APEX2 compared to TOMM70-APEX2. To investigate this further, we subjected the significantly enriched proteins in the differential TOMM20 vs TOMM70 proximitome to a STRING-based network analysis.^38^ The majority (43%) of these proteins in the TOMM20-APEX2 proximitome are overrepresented for cytoplasmic localization with multiple of them annotated as RBP (Figure 4B; up). STRING analysis further reveals that most of the processes involving the enriched RBPs are interconnected, supporting our hypothesis that TOMM20-APEX2 specifically biotinylates a distinct and functionally related group of RBPs and translation factors that are in its close proximity, rather than doing so randomly. We further checked if these proteins were also enriched in our other comparisons of proximitomes (TOMM20-APEX2 vs -DOX, TOMM20-APEX2 vs APEX2-NES, and TOMM20-APEX2 vs Mito-APEX2; Supporting Table S11), which revealed that many RBPs and translation factors including PAIP1, PABPCL, and EIF5 were enriched in the TOMM20 proximitome versus these other ones. In contrast, STRING analysis of the TOMM70-APEX2 revealed that most of the proteins that are more enriched compared to TOMM20-APEX2 are linked to membrane surrounded organelles (Figure 4B; down).

The identification of several RBPs or proteins involved in translation in our enriched proteomes prompted us to investigate how the proximitomes of TOMM20- and TOMM70-APEX2 changes in the presence of the translation inhibitor puromycin. For that, we included three replicates of puromycin treated (+puro) samples for TOMM20- and TOMM70-APEX2 expressing cells. For treatment, we chose a 30 min window and 200 μM concentration of puromycin since a similar treatment had revealed only little change in the overall proteome in HEK293 cells.^16,27^ Puromycin treatment (+puro) of TOMM20-APEX2 expressing cells resulted in surprisingly little change in the associated proteome with only few proteins more (PRTEDC1) or less enriched (FANC1, CHERP, and FADS1) after treatment (Supporting Figure S8A; Table S12). However, none of these proteins have an obvious connection to each other, to RNA, or to mitochondrial function. A similar observation was made upon treatment of TOMM70-APEX2 cells with puromycin although the number of less or more enriched proteins was slightly larger (Supporting Figure S8B; Table S13). In a subsequent step we compared the differential proximitome of TOMM20 vs TOMM70 (both after treatment with puromycin and normalized against uninduced controls) and focused especially on proteins annotated by GOBP as being involved in translation and mRNA processing. This analysis revealed in total 258 proteins, of which 11 were enriched in the TOMM20-APEX2 proximitome after puromycin treatment compared to that of TOMM70-APEX after exposure to puromycin (Figure 4C; Supporting Table S14). These include translation initiation and elongation factors (EIF4B, EIF4H, EEF1D), ribosomal proteins (RPS5, RPL10A), and RBPs that are primarily involved in controlling mRNA stability. Among the latter proteins are SYNJ2BP, a MOM-localized RBP that binds to mRNAs encoding mitochondrial proteins, mitigates the effects of translation stress, and has been linked to the local translation at the mitochondrial surface;^27^ PAIP1, a translational coactivator interacting with polyA-binding protein PABP;^49^ and finally SNCA, an RBP that forms an amphipathic helix, similar to the MTS motifs,^50^ and is a known interactor of TOMM20.^50,51^ The enrichment of RBPs that are involved in mRNA stability regulation in the TOMM20-APEX2 data set after translation inhibition suggests that TOMM20, via interacting with these proteins might have a more mRNA-protective role than TOMM70.

In a similar analysis as before (Figure 4B), the significantly enriched proteins in the TOMM20-APEX2 (+puro) (Figure 4D; up) and TOMM70-APEX2 (+puro) (Figure 4D; down) data sets were subjected to STRING-based network analysis. For the TOMM20-APEX2 proximitome, we identified a cluster of proteins annotated as translation regulators. This group of proteins includes a RBP (PAIP1), translation factors (EIF4B, EIF4H, EEF1D), and ribosomal protein (RPS4), suggesting that components of the translation machinery are still proximal to TOMM20 upon puromycin inhibition.

### Structural Prediction, Coimmunoprecipitation, and Super-Resolution Microscopy Verify an Interaction of TOMM20 and SYNJ2BP

We chose the RBP SYNJ2BP to verify if the observed enrichment of RBPs in the TOMM20-APEX2 proximity reflects its direct interactions with this TOM complex component. Structure prediction by AlphaFold 3 ^40^ for the cytosolic domains of both proteins (aa 25–145 of TOMM20 and aa 1–117 of SYNJ2BP) suggests a cytoplasmic interaction interface of the two proteins (ipTM score of 0.47; Figure 5A,B) with multiple potentially interacting residues within a 8 Å threshold distance (Figure 5A,B). Multiple TOMM20 residues VAL42, GLN43, PHE46, and LEU47 interact with amino acids of SYNJ2BP located in its PDZ domain (Supporting Table S15). In contrast, AlphaFold was unable to predict a high-confidence model for the cytoplasmic domains of SYNJ2BP and TOMM70 (ipTM score = 0.32). None of the interacting residues were identified in 8 Å distance between both proteins (Supporting Figure S9). The predicted interaction of SYNJ2BP and TOMM20 was verified in coimmunoprecipitation experiments using isolated mitochondria, where the V5-tagged TOMM20-APEX2 not only copurified with an established interactor (TOMM22) but also with SYNJ2BP, independent of the presence of puromycin (Figure 5C). This finding of an interaction of both proteins is supported by super-resolution microscopy of endogenous TOMM20 and SYNJ2BP, which reveals a high degree of positional overlap of both proteins (Figure 5D).

**Figure 5:**
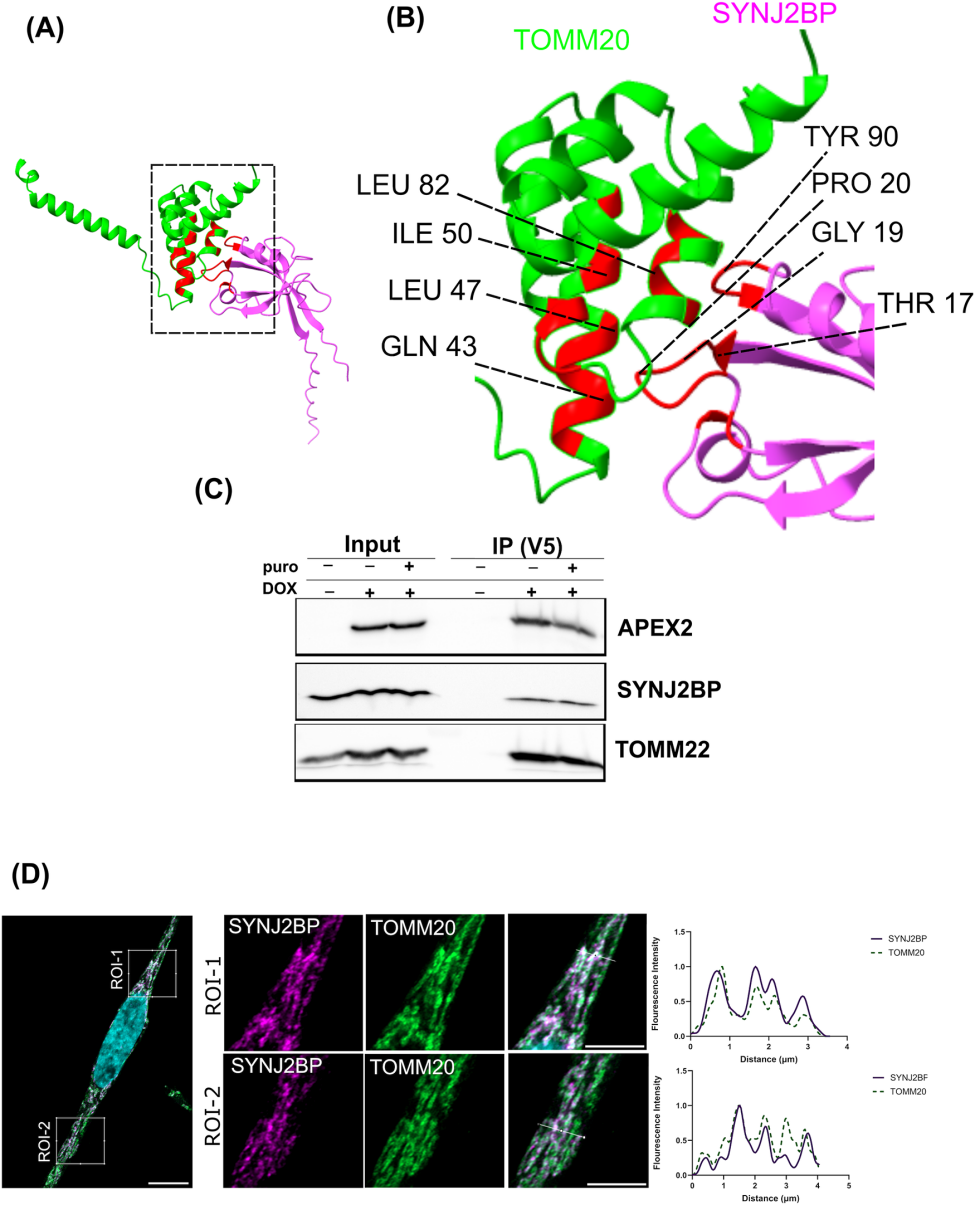
Interaction of TOMM20 and SYNJ2BP. (A) Structural modeling predicts specific molecular interactions between TOMM20 and SYNJ2BP. AlphaFold3 was run on the cytoplasmic domains of human TOMM20 (aa 25–145) and SYNJ2BP (aa 1–117). The ipTM score for the TOMM20-SYNJ2BP interaction is 0.47. The distance threshold for AlphaFold contacts visualized by ChimeraX is 8 Å. (B) Close-up view showing highlighted residues (red) predicted to interact between the two proteins. (C) Western blot showing the coimmunoprecipation of endogenous SYNJ2BP with TOMM20-APEX2 using anti-V5 beads that capture the fusion protein. (D) Super resolution microscopy images showing colocalization of SYNJ2BP and TOMM20. Wildtype HeLa 11ht cells were fixed and immunolabeled with antibodies directed against TOMM20 (green) and SYNJ2BP (magenta). Each confocal image represents a merged image of green and magenta channels, with two ROIs selected in the cell. Nuclei were stained with DAPI (cyan). Scale bar, 10 μm. Each ROI is a zoomed-in portion of the cell in the middle. Scale bars, 5 μm. Scan intensity profiles represent the overlapping fluorescence signals of SYNJ2BP and TOMM20.

In summary, our quantitative proteomic approach using differential proximitomes supports the hypothesis that the human TOMM20 and TOMM70 proteins interact with unique sets of proteins, that TOMM20 shows the propensity of interaction with RBPs and that it partially remodels its proteome in response to translation stress.

## Discussion

Proximity labeling approaches have been widely used to study the mitochondrial proximal proteome and transcriptome.^16,27,32,52^ In most of these studies, the proximity ligase was targeted to the MOM by fusing it to a targeting peptide from the MAVS protein.^27,32,53^ While this method enables the targeting of APEX2 or TurboID enzymes to the MOM, it does not reveal whether the identified associated proteins are functionally related to the TOM complex that controls mitochondrial protein import. To address this issue directly, we employed proximity labeling based on APEX2 and quantitative mass spectrometry to examine the presence of mRNA-interacting proteins near the TOM complex at the MOM and compared the proximitomes of the mammalian TOMM20 and TOMM70 proteins in HeLa cells.

We demonstrated the feasibility of our approach by demonstrating an enrichment of MOM proteins in proximity to the TOM complex, especially when comparing TOMM-APEX2 proximitomes with that of cytoplasmic or mitochondrial matrix localized APEX2. Particularly in case of TOMM20-APEX2, we identified, besides expected MOM components like MTARC1 (a MOM-localized oxidoreductase), OCIAD1 (OCIA domain-containing protein 1), CISD1 (a redox active mitochondrial protein with an iron–sulfur domain), RMDN3 (a regulator of microtubule dynamics), BCL2L13 (a BCL2-like protein), and CYB5R3 (NADH-cytochrome b5 reductase 3), several RNA-binding proteins. These include SYNJ2BP and MAVS that had already previously been linked to the MOM.^27,32^

In a related study, the proximitome of human TOMM20 was recently captured in HCT116 cells by tagging it with the miniTurbo biotin ligase.^46^ 315 proteins out of 700 enriched in our TOMM20-APEX2 proximitome were also enriched in TOMM20 miniTurboID proximitome (Supporting Figure S10; Table S16). These proteins include 71 mitochondrial proteins but also 50 translation-related proteins. In a recent copurification approach, Özdemir et al.^54^ immunoprecipiated a Flag-tagged TOMM20 to identify bound proteins. In contrast to the two proximity labeling approaches, a larger number of mitochondrial proteins (177 out of 360) were identified to interact with TOMM20 but It was not revealed if these were transport intermediates transiently interacting with the TOMM20 receptor. Comparing their data set with the one obtained in this study reveals an overlap of 92 identified mitochondrial proteins. However, the number of overlapping MOM proteins found in these two studies (27 proteins) is larger compared to that identified in both proximity labeling approaches, whereas the number of translation-related factors is similar. Interestingly, 14 of the 104 proteins identified in all three approaches can be attributed to RBPs or RNA-related functions (Supporting Figure S10; Table S16), supporting the idea of translation processes in the vicinity of the TOM complex.

Proximity to RBPs seems to be more evident for TOMM20-APEX2 in contrast to TOMM70-APEX2 since it appears not only to be close to MOM-associated (SYNJ2BP) but also cytoplasmic RBPs like PAIP1, KSHRP, and PABPC4L, and components of the translation machinery including ribosomal proteins or the translation initiation factor EIF5. This enrichment of RBPs and components of the translation machinery not only corroborates the hypothesis of localized translation at the MOM^18,22,23^ but also suggests that TOMM20 rather than TOMM70 might play a role in localized translation of nuclear encoded mitochondrial mRNAs or cotranslational import of their encoded proteins.

The remodeling of its proximitome during inhibition of translation suggests that TOMM20 might play a role in preserving cellular homeostasis during translation stress by retaining translation-related proteins at the MOM. This is especially evident from the enrichment of RPBs involved in mRNA stability like SYNJ2BP, SNCA, or PAIP1 even under translation stress conditions. SYNJ2BP has previously been identified as a key regulator to safeguard specific nuclear-encoded mitochondrial transcripts during translation stress.^27^ It anchors these transcripts under stress recovery which might facilitate their local translation and import, thereby maintaining OXPHOS activity and mitochondrial function.^27^ Our data suggests that TOMM20 directly interacts with SYNJ2BP. We also found that, under translation arrest, components of the translation machinery like EIF4B, EIF4H, EEF1D, or ribosomal proteins, are still enriched in the TOMM20-APEX2 but not the TOMM70-APEX2 proximitome. This could indicate a SYNJ2BP-mediated localized translation in proximity of the TOMM20 receptor. α-Synuclein (SNCA), a noncanonical RBP is a known interactor of TOMM20,^50,51^ and TOMM20 overexpression reportedly rescued α-Synuclein induced dopaminergic neurodegeneration in Parkinson’s disease patients.^55^ Interestingly, SYNJ2BP has also been linked to Parkinsons disease, suggesting that both RBPs contribute to mitochondrial health.^56^ Finally, PAIP1 interacts with key translation factors and modulates the stability and translation of mRNAs.^49,57–59^ The presence of these RBPs in the TOMM20 proximitome under translation inhibition conditions could reflect their direct role as safeguards against translation stress. In the future, the integration of similar APEX2-based proximity labeling approaches of other TOM complex subunits or TOM-associated proteins like the SAM complex^60^ with the presented data could reveal a more detailed view of the complex interactions at the MOM and provide further insights into the processes of localized translation at the MOM and its contribution to mitochondrial import.

## Supplementary Material





## Data Availability

The mass spectrometry proteomics data have been deposited in the ProteomeXchange Consortium via the PRIDE partner repository39 with the data set identifier PXD057097 (doi:10.6019/PXD057097).

## Notes

Expression of TOMM20-APEX2 or TOMM70-APEX2 do not interfere with mitochondrial function in the HeLa cells (Figure S1); protein biotinylation is mediated by TOMM20- or TOMM70-APEX2 fusion proteins (Figure S2); expression of cytoplasmic (APEX2-NES) and mitochondrial matrix localized (mito-APEX2) fusion proteins (Figure S3); compartment specific local biotinylation mediated by APEX2-NES and mito-APEX2 (Figure S4); combining replicates from two independent experiments mildly increases protein identification without altering relative quantification (Figure S5); quantitative analysis of TOMM20- and TOMM70-APEX2 proximitomes (Figure S6); proximitomes of TOMM20-APEX2 and TOMM70-APEX2 against Mito-APEX2 proximitomes (Figure S7); effect of puromycin on TOMM20- and TOMM70-APEX2 proximitomes (Figure S8); lack of AlphaFold based interaction prediction between TOMM70 and SYNJ2BP (Figure S9); comparison of results from Meurant et al. and Oezdemir et al. highlights shared candidates (Figure S10); list of plasmids used in study (Table S1); list of antibodies used in study (Table S2) (PDF)
Pearson correlation table (Table S3); proteins identified in TOMM20-APEX2 vs -DOX proximitome (Table S4); proteins identified in TOMM70-APEX2 vs -DOX proximitome (Table S5); proteins identified in TOMM20-APEX2 vs APEX2-NES proximitome (Table S6); proteins identified in TOMM70-APEX2 vs APEX2-NES proximitome (Table S7); proteins identified in TOMM20-APEX2 vs Mito-APEX2 proximitome (Table S8);proteins identified in TOMM20-APEX2 vs Mito-APEX2 proximitome (Table S9); proteins identified in TOMM20-APEX2 vs TOMM70-APEX2 proximitome (Table S10); overlapping of TOMM20-APEX2 candidates among different proximitomes (Table S11); proteins identified in TOMM20-APEX2 vs TOMM20-APEX2 (+puro) proximitome (Table S12); proteins identified in TOMM70-APEX2 vs TOMM70-APEX2 (+puro) proximitome (Table S13); proteins identified in TOMM20-APEX2 (+puro) vs TOMM70-APEX2 (+puro) proximitome (Table S14); predicted interacting residues between TOMM20 and SYNJ2BP predicted in AlphaFold model (Table S15); comparison of results from Meurant et al. and Oezdemir et al. highlights shared candidates (Table S6) (ZIP)
