# Supplementary material for: Proximity Labeling Reveals RNA-Binding Proteins Associating with the Human Mitochondrial Import Receptor TOMM20
Source: J Proteome Res. 2025 Dec 22;25(2):1055–70. doi: 10.1021/acs.jproteome.5c00905 (PMC12888002; doi:10.1021/acs.jproteome.5c00905)
Supplement: Supplementary file 1 [file pr5c00905_si_001.pdf]

**Supporting Information**

***Proximity labeling reveals RNA-binding proteins associating with the human mitochondrial import receptor TOMM20***

Saira Akram[1], Katharina I. Zittlau[2,3], Karan Sharma[4], Julia C. Fitzgerald[4], Nisha Rafiq[1], Boris Maček[2], Ralf-Peter Jansen[1,*]

1 Interfaculty Institute of Biochemistry, University of Tübingen, 72076 Tübingen, Germany
2 Proteome Center Tübingen, University of Tübingen, 72076 Tübingen, Germany
3 Department of Biomolecular Sciences, Weizmann Institute of Science, Rehovot 76100, Israel
4 Department of Neurodegeneration, Hertie Institute for Clinical Brain Research, Centre for Neurology, Faculty of Medicine, University of Tübingen, 72076 Tübingen, Germany

* Corresponding author:
Interfaculty Institute of Biochemistry, University of Tübingen
Auf der Morgenstelle 34, 72076 Tübingen, Germany
ralf.jansen@uni-tuebingen.de

*Table of Contents*


| | |
|---|---|
| Supplementary Figure 1 | Expression of TOMM20-APEX2 or TOMM70-APEX2 do not interfere with mitochondrial function in the HeLa cells |
| Supplementary Figure 2 | Protein biotinylation is mediated by TOMM20- or TOMM70-APEX2 fusion proteins. |
| Supplementary Figure 3 | Expression of cytoplasmic (APEX2-NES) and mitochondrial matrix localized (mito-APEX2) fusion proteins. |
| Supplementary Figure 4 | Compartment specific local biotinylation mediated by APEX2-NES and mito-APEX2 |
| Supplementary Figure 5 | Combining replicates from two independent experiments mildly increases protein identification without altering relative quantification. |
| Supplementary Figure 6 | Quantitative analysis of TOMM20- and TOMM70-APEX2 proximitomes |
| Supplementary Figure 7 | Proximitomes of TOMM20-APEX2 and TOMM70-APEX2 against Mito-APEX2 proximitomes |
| Supplementary Figure 8 | Effect of puromycin on TOMM20- and TOMM70-APEX2 proximitomes. |
| Supplementary Figure 9 | Lack of AlphaFold based interaction prediction between TOMM70 and SYNJ2BP |
| Supplementary Figure 10 | Comparison of results from Meurant et al. and Oezdemir et al. highlights shared candidates |
| Supplementary Table S1 | List of plasmids used in study |
| Supplementary Table S2 | List of antibodies used in study |

| | |
|---|---|
| Supplementary Table S3.xlxs | Pearson correlation table |
| Supplementary Table S4.xlxs | Proteins identified in TOMM20-APEX2 vs -DOX proximitome |
| Supplementary Table S5.xlxs | Proteins identified in TOMM70-APEX2 vs -DOX proximitome |
| Supplementary Table S6.xlxs | Proteins identified in TOMM20-APEX2 vs APEX2-NES proximitome |
| Supplementary Table S7.xlxs | Proteins identified in TOMM70-APEX2 vs APEX2-NES proximitome |
| Supplementary Table S8.xlxs | Proteins identified in TOMM20-APEX2 vs Mito-APEX2 proximitome |
| Supplementary Table S9.xlxs | Proteins identified in TOMM70-APEX2 vs Mito-APEX2 proximitome |
| Supplementary Table S10.xlxs | Proteins identified in TOMM20-APEX2 vs TOMM70-APEX2 proximitome |
| Supplementary Table S11.xlxs | Overlapping of TOMM20-APEX2 candidates among different proximitomes |
| Supplementary Table S12.xlxs | Proteins identified in TOMM20-APEX2 vs TOMM20-APEX2 (+puro) proximitome |
| Supplementary Table S13.xlxs | Proteins identified in TOMM70-APEX2 vs TOMM70-APEX2 (+puro) proximitome |
| Supplementary Table S14.xlxs | Proteins identified in TOMM20-APEX2 (+puro) vs TOMM70-APEX2 (+puro) proximitome |

| | |
|---|---|
| Supplementary Table S15.xlxs | Predicted interacting residues between TOMM20 and SYNJ2BP predicted in AlphaFold model |
| Supplementary Table S16.xlxs | Table showing comparison of results from Meurant et al. and Oezdemir et al. highlights shared candidates |

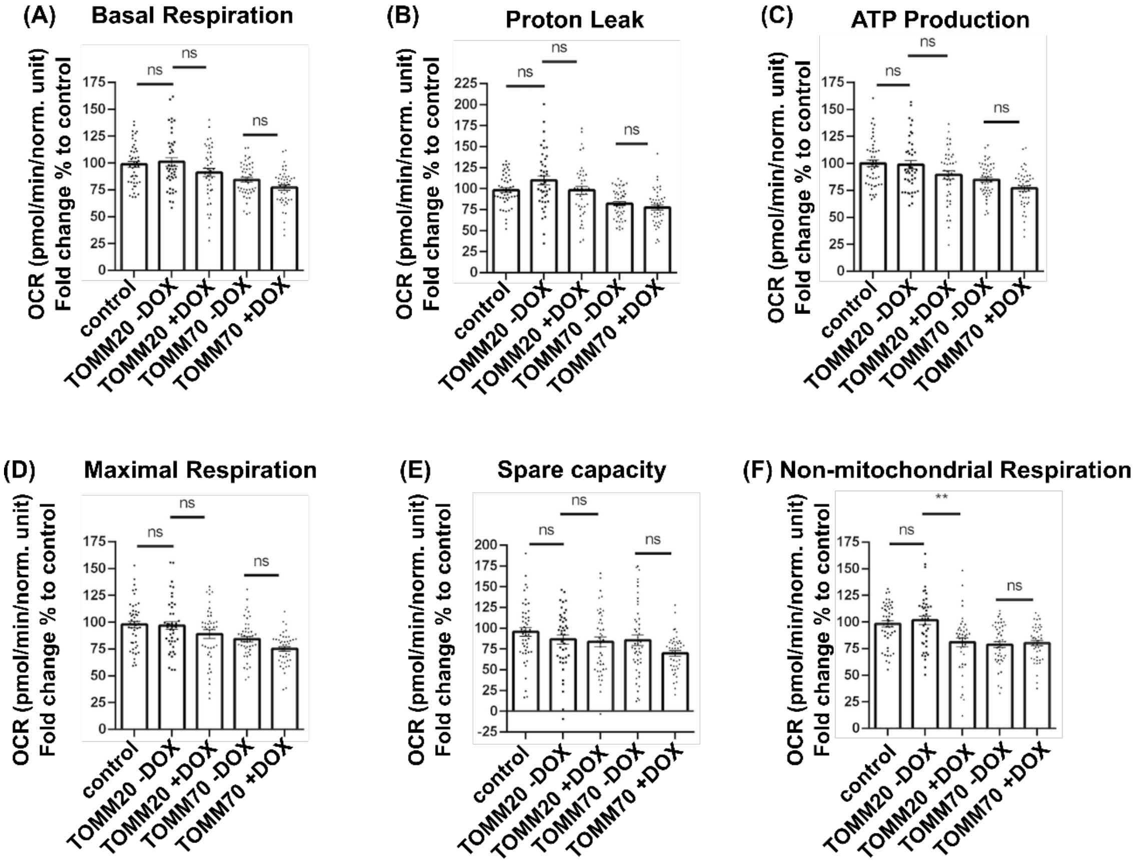


**Supplementary Figure S1. Expression of TOMM20-APEX2 or TOMM70-APEX2 do not interfere with mitochondrial function in the HeLa cells.**
Seahorse based mitochondrial stress test to measure Oxygen Consumption Rate (OCR) and Extracellular Acidification Rate (ECAR) in HeLa 11ht cells (control), and TOMM20-APEX2 and TOMM70-APEX2 expressing cells with (+) and without (-) the DOX induction. The OCR values obtained were used for the quantification of **(A)** Basal respiration, **(B)** Proton leak, **(C)** ATP Production, **(D)** Maximal Respiration, **(E)** Spare capacity and **(F)** Non-mitochondrial respiration (n=11-16 from 3 independent biological replicates). The error bars show the standard error of mean (SEM). For statistical analysis with more than two samples, an ordinary one-way ANOVA was used with Tukey's multiple comparisons tests.

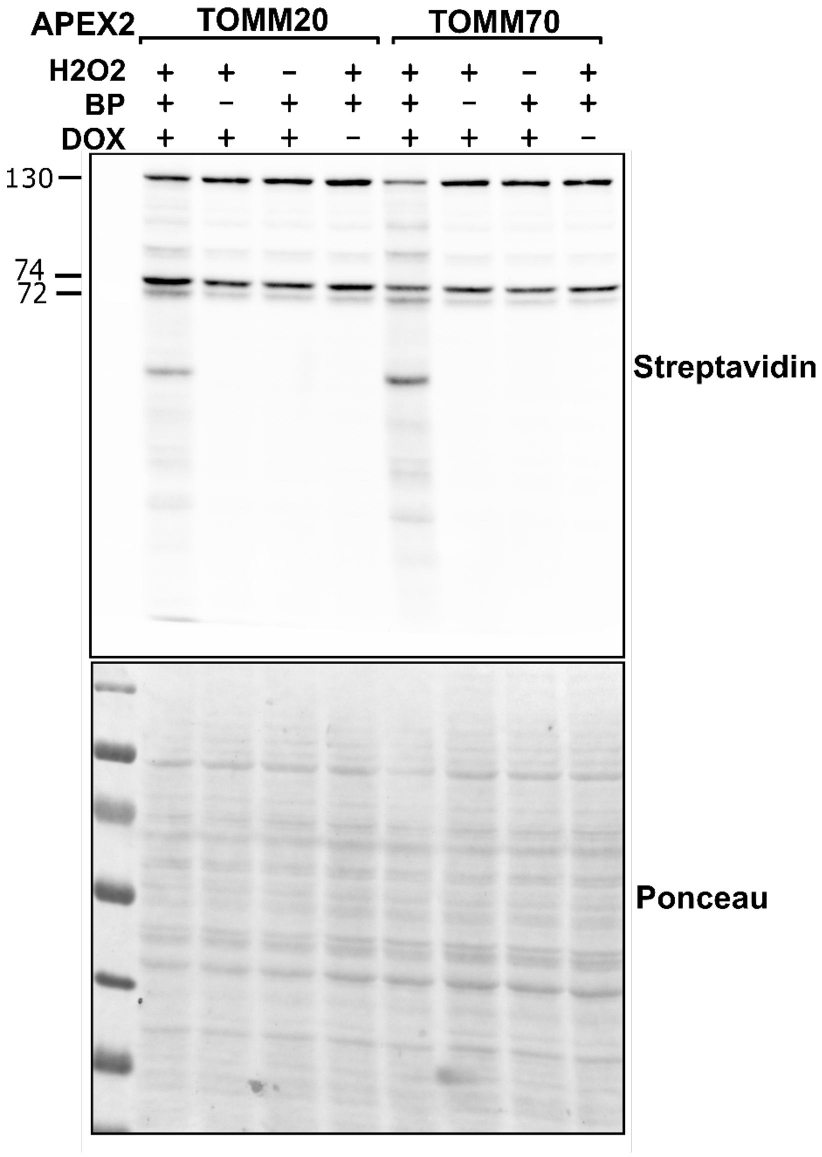


**Supplementary Figure S2. Protein biotinylation is mediated by TOMM20- or TOMM70-APEX2 fusion proteins.**

Western blot analysis of cell lysates to confirm the APEX2-mediated biotinylation in Hela cells stably expressing the indicated APEX2 fusion proteins. The blot is stained either with Streptavidin-HRP conjugate to detect biotinylated proteins (upper part) or with Ponceau S (lower part). Biotinylation depends on the presence of both biotin phenol (BP) and $H_2O_2$. Numbers on the left refer to protein mass in kDa. Omission of BP or $H_2O_2$or lack of APEX2 fusion protein expression results only in detection of endogenous biotin-containing proteins including 3-methylcrotonyl-CoA carboxylase alpha (MCCC1; ~72 kDa), propionyl-CoA carboxylase alpha (PCCA; ~ 74 kDa), and pyruvate carboxylase (PC; ~130 kDa).

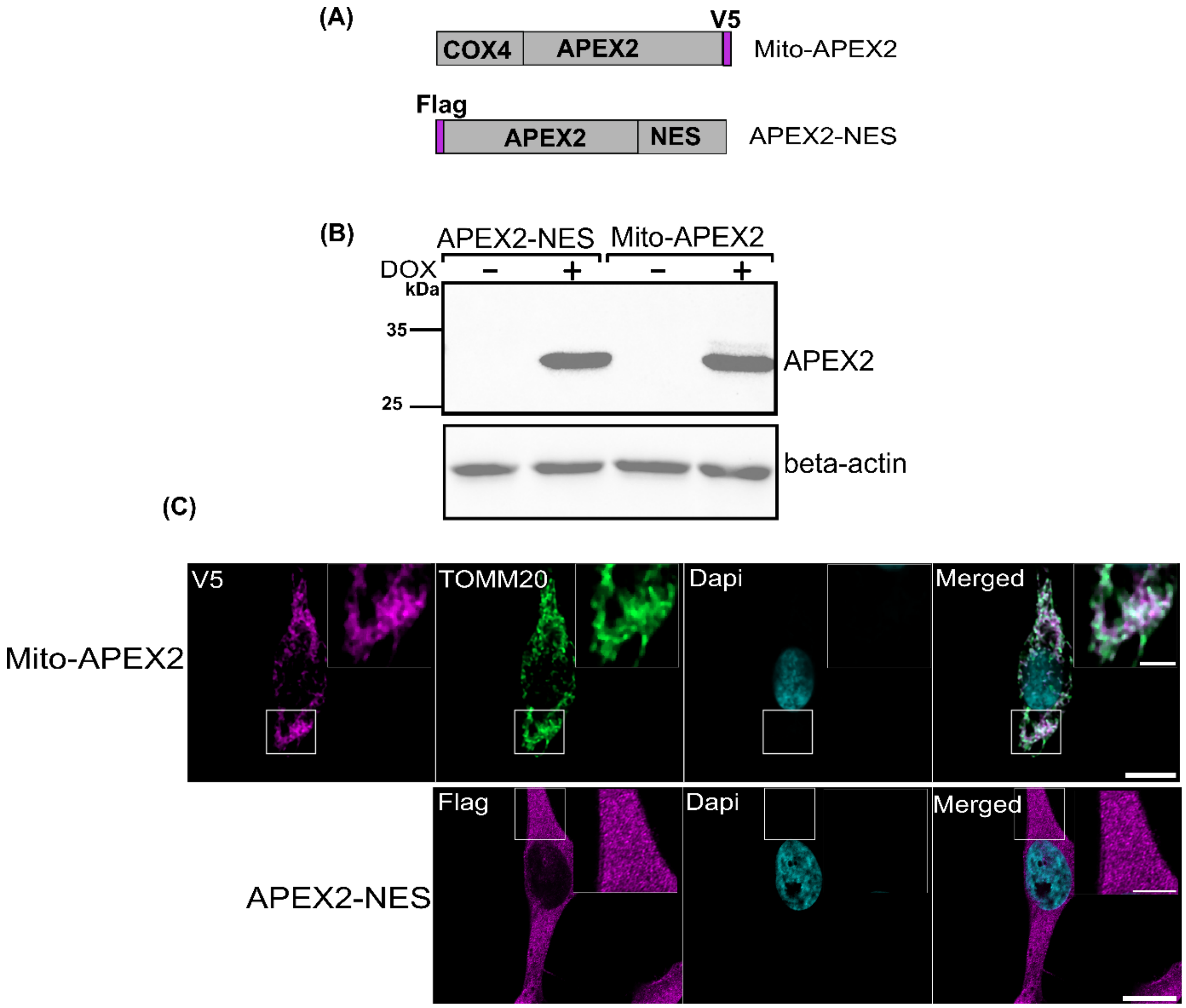


**Supplementary Figure S3. Expression of cytoplasmic (APEX2-NES) and mitochondrial matrix localized (mito-APEX2) fusion proteins.**
**(A)** Domain structures of fusion proteins stably expressing APEX2 in various cellular compartments. Mito-APEX2 harnesses a 1-24 amino-acid sequence from Mitochondrial matrix resident 'COX4' protein to localize APEX2 in the mitochondrial matrix. APEX2-NES utilizes a nuclear export signal (NES) to target APEX2 to the cytoplasm. **(B)** Western blot analysis of whole cell lysate of cells stably expressing, and APEX2-NES constructs. Cells are either not or induced with DOX for 24 hours prior to lysis. APEX2 containing fusion proteins are analyzed with APEX2 antibody. Beta-actin is used as loading control. **(C)** Confocal fluorescence imaging confirming the cellular localization of stably expressing Mito-APEX2 and APEX2-NES fusion proteins. Cells are induced with DOX for 24 hours, immunolabeled with antibodies directed against V5 to detect Mito-APEX2 proteins (magenta), or flag to visualize APEX2-NES fusion protein (magenta). (Scale bar: 10 μm; inset: 5 μm).

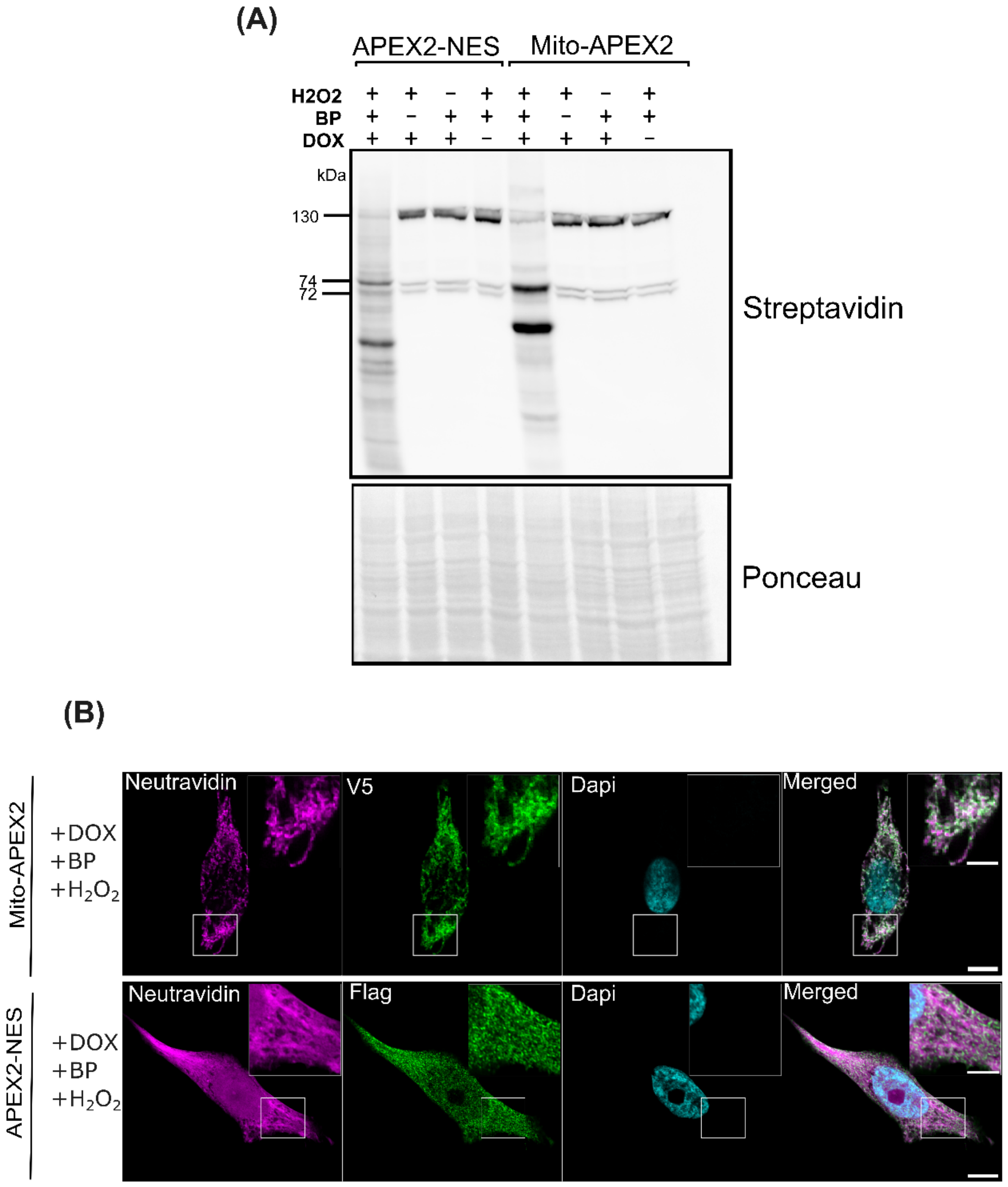


**Supplementary Figure S4. Compartment specific local biotinylation mediated by APEX2-NES and Mito-APEX2.**
**(A)** Western blot analysis of cell lysates to confirm the APEX2-mediated biotinylation in the cells stably expressing Mito-APEX2 and APEX2-NES constructs. Biotinylated proteins are probed by Streptavidin-HRP conjugate (upper part), Ponceau S staining is shown below. Omission of BP or $H_2O_2$ or lack of APEX2 fusion protein expression results only in the detection of endogenous biotinylated proteins at 130, 74 and 72 kDa. **(B)** Confocal fluorescence imaging of APEX2-mediated biotinylation in HeLa cells stably expressing the Mito-APEX2 and APEX2-NES constructs. Following 24 hours of DOX induction, cells are subjected to biotinylation with biotin phenol (BP) and $H_2O_2$ and stained with either anti-V5 or anti-Flag antibodies to confirm the expression of the indicated APEX2 fusion proteins (green), while Alexa-647 labeled neutravidin is utilized to stain biotinylated proteins (magenta). Nuclei are stained with DAPI (cyan). (Scale bar: 10 μm; scale bar in inset: 5 μm).

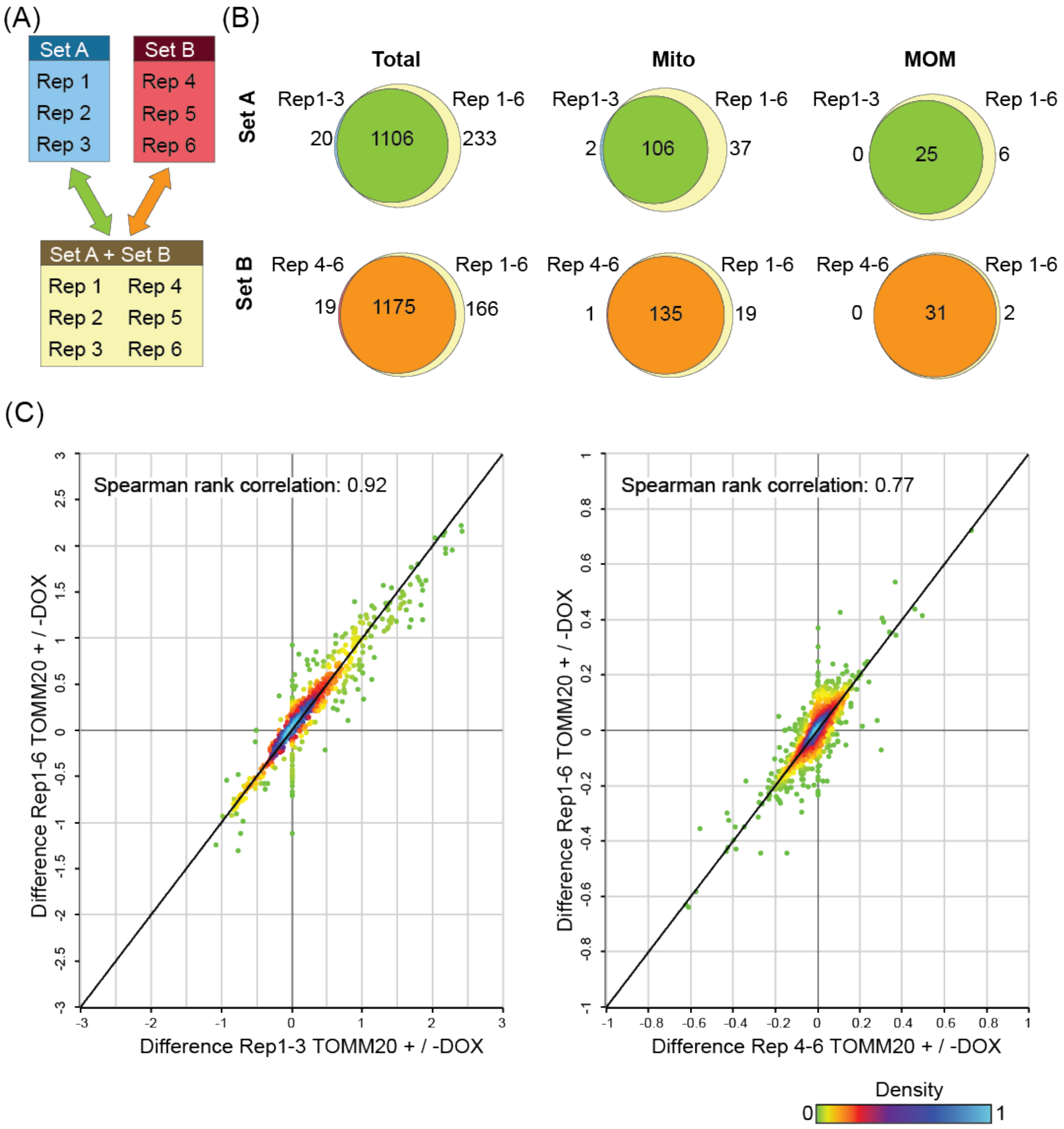


**Supplementary Figure S5. Combining replicates from two independent experiments mildly increases protein identification without altering relative quantification.**
**(A)** TOMM20-APEX data were re-processed either separately as Set A (Replicates 1–3) and Set B (Replicates 4–6) or combined (Replicates 1–6). **(B)** The majority of proteins are consistently identified across all processing strategies, regardless of the number of replicates used. Shown are total proteins identified, as well as subsets annotated with mitochondrial and mitochondrial outer membrane (MOM) localization. **(C)** Spearman rank correlations of protein-level abundance differences between TOMM20-APEX and TOMM20-APEX (-DOX) conditions demonstrate high reproducibility between individual sets and the combined set. Density plots illustrate the distribution of protein differences, and Spearman correlation coefficients are indicated.

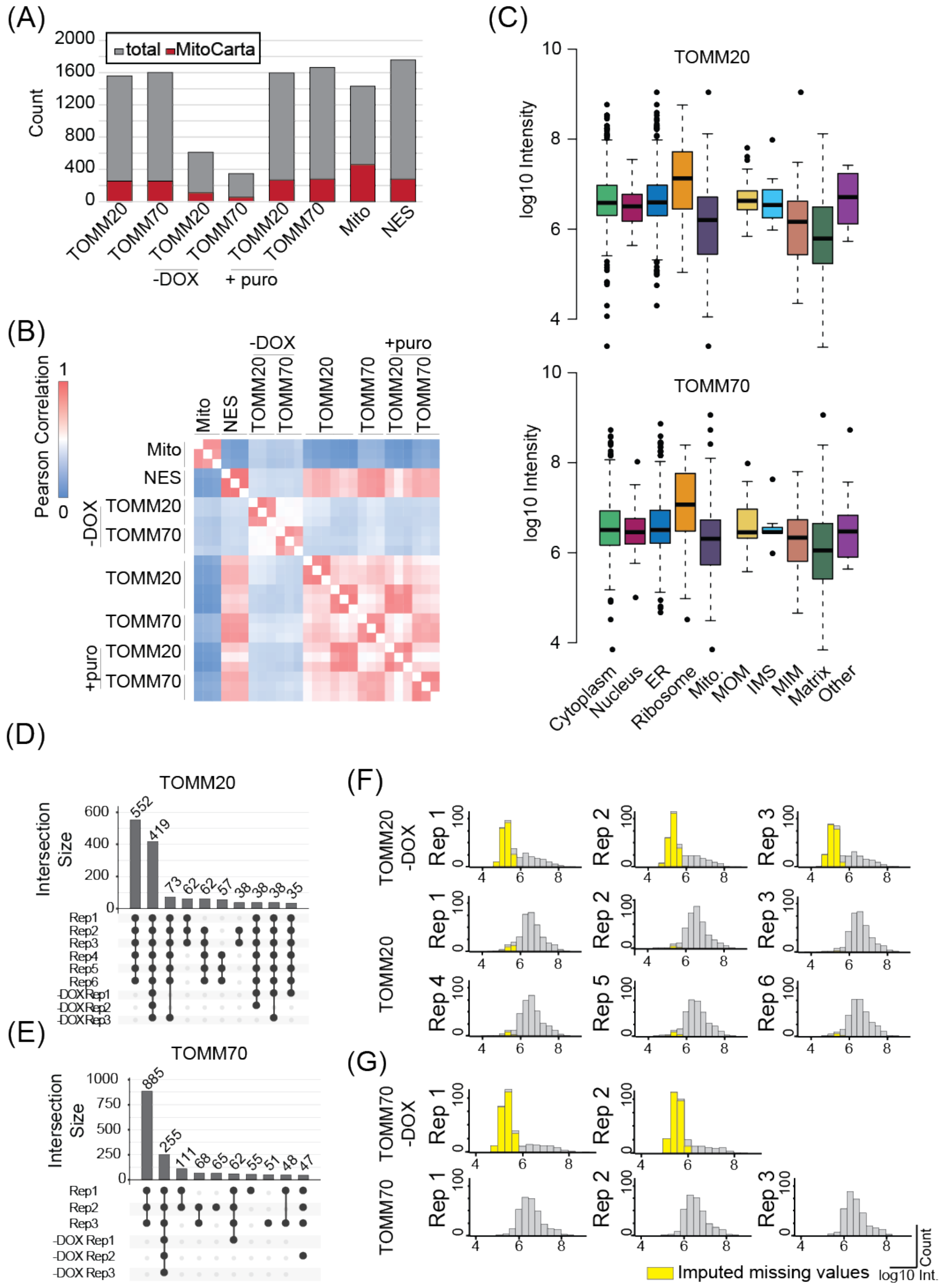


**Supplementary Figure S6. Quantitative analysis of TOMM20- and TOMM70-APEX2 proximitomes**

**(A)**Total identification of proteins (grey) and proteins annotated for mitochondrial localization (red) based on MitoCarta3.0. Count of proteins based on quantification in minimum 3 out of 6 replicates for TOMM20-APEX and 2 out of 3 replicates for remaining samples. Puro samples correspond to puromycin treated samples (also see figure 4) (**B)** Correlation between replicates based on Pearson correlation. **(C)** Box plots showing the log-transformed intensity

of organelle-annotated proteins identified in TOMM20-APEX2 (upper pannel) and TOMM70-APEX2 (lower pannel). (**D)** Upset plot of overlapping proteins identified between replicates of TOMM20-APEX2 and controls (-DOX TOMM20-APEX2). (**E)** Upset plot of overlapping proteins identified between replicates of TOMM70-APEX2 and controls (-DOX TOMM70-APEX2). Imputation of missing values (yellow bars) showing mostly unidentified low abundant proteins replaced from normal distribution for -DOX TOMM20-APEX **(F)** and -DOX TOMM70-APEX2 **(G)**.

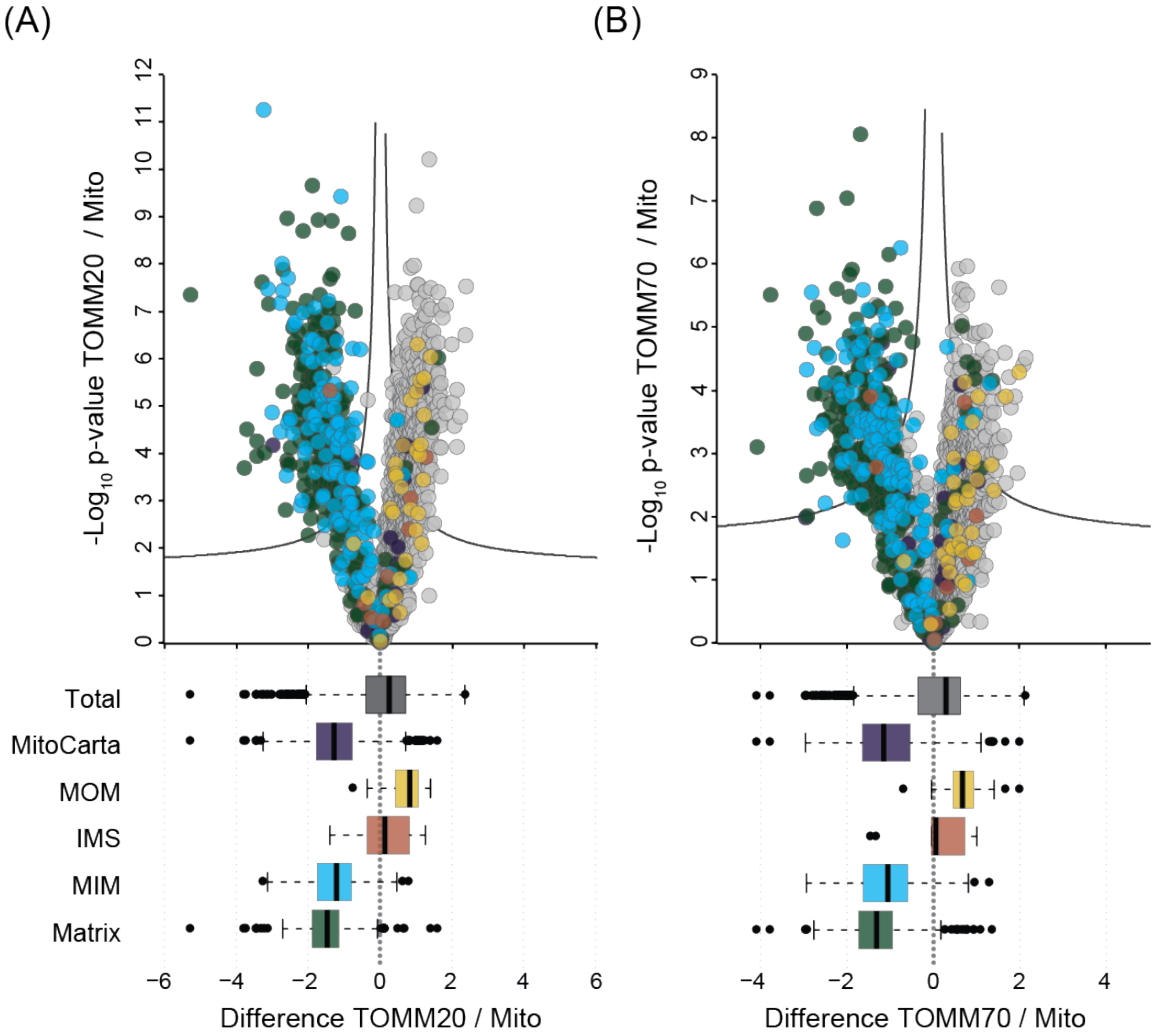


**Supplementary Figure S7. Comparison of TOMM20-APEX2 and TOMM70-APEX2 proximitomes with that of Mito-APEX2 reveals enrichment for MOM and IMS over matrix and MIM proteins.**
Volcano plots for TOMM20-APEX2 (A) and TOMM70-APEX2 (B) against Mito-APEX2 proximitomes. Highlighted are proteins annotated for their respective submitochondrial localization based on MitoCarta3.0. Boxplots show the distribution of total proteins compared to proteins annotated for mitochondrial, and mitochondrial sub localization for TOMM20- (left) and TOMM70-APEX2 (right) against Mito-APEX2. Indicated are thresholds for significantly enriched proteins (p-value 5%, Difference 3).

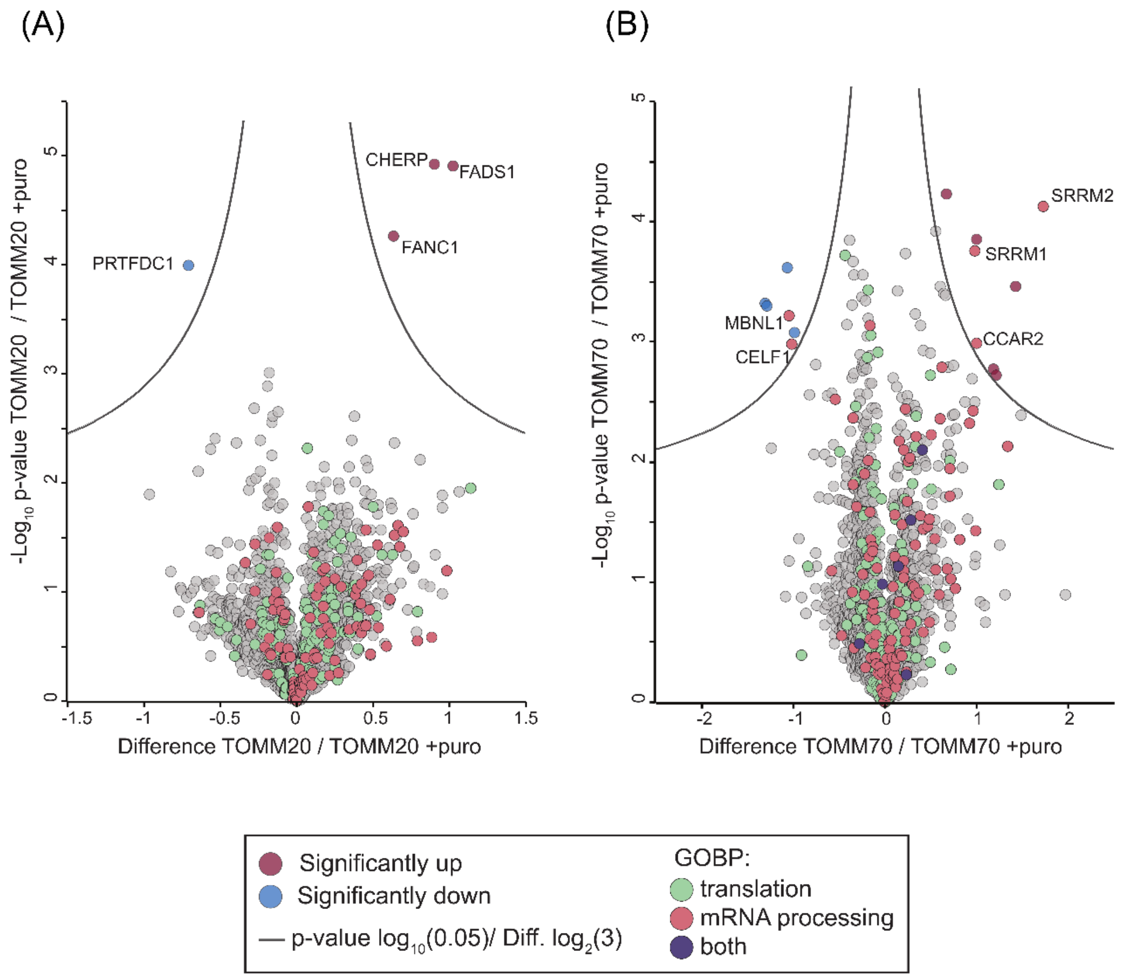


**Supplementary Figure S8. Effect of puromycin on TOMM20- and TOMM70-APEX2 proximitomes**.
(**A**) Volcano plots for TOMM20-APEX2 proximitome against TOMM20-APEX2 proximitome (+puro). (**B**) Volcano plots for TOMM70-APEX2 proximitome against TOMM70-APEX2 proximitome (+puro). Highlighted are the proteins annotated (based on GOBP) for translation and mRNA processing.

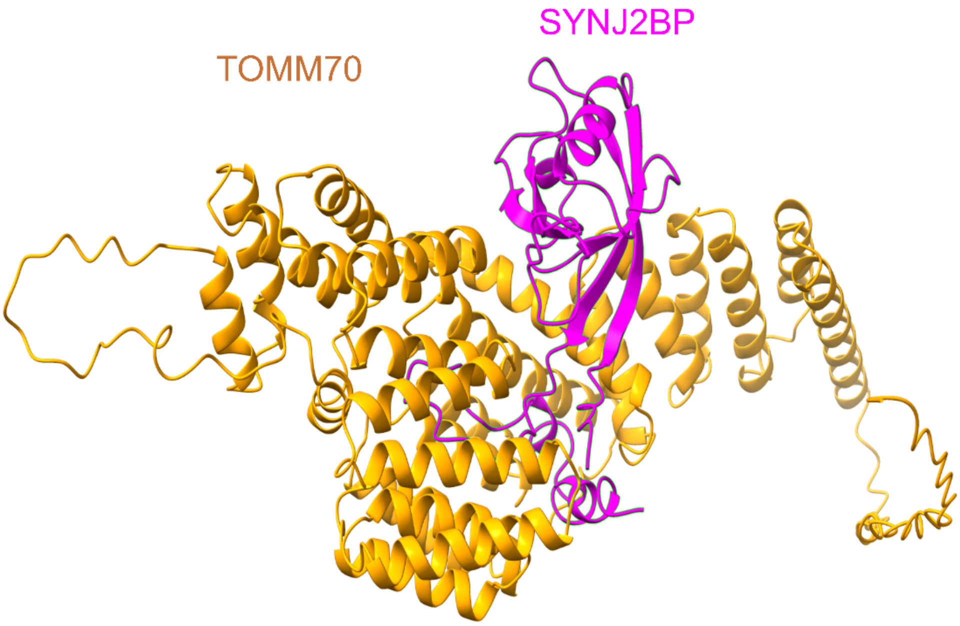


**Supplementary Figure S9.**

**AlphaFold based interaction prediction between TOMM70 and SYNJ2BP.**
Structural modeling shows none of the interacting residues between human TOMM70 and SYNJ2BP proteins. AlphaFold3 generated ipTM score of 0.32 when run on the cytoplasmic domains of human TOMM70 (aa 60-608) and SYNJ2BP (aa 1-117). None of the residues between the proteins are at interface and predicted to be interacting within 8 Å distance threshold.

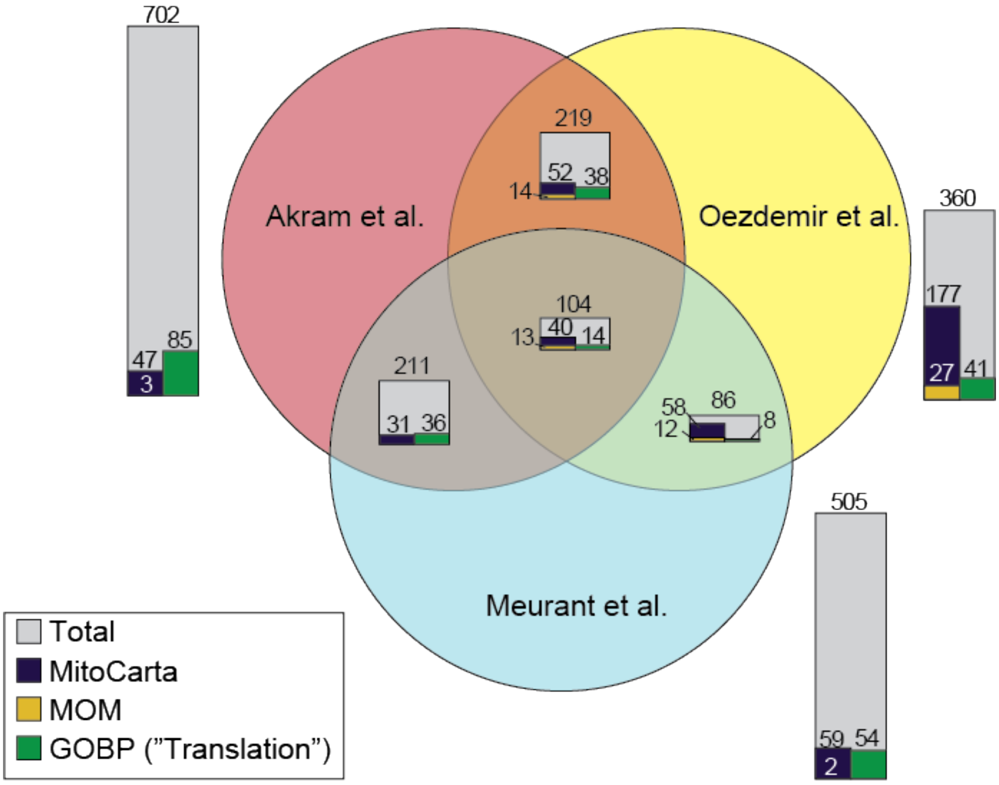


**Supplementary figure S10.**

**Comparison of results from Meurant et al.**[36] **and Oezdemir et al.**[46] **highlights shared candidates.** 14 enriched GOBP (translation) proteins are MRPL45, EIF3E, EEF2, GSPT1, EEF1D, RPS27A, EIF3A, CAPRIN1, KARS, LARP1, EIF3M, UPF1, MRPL37, MRPS30 **(Suppl. Table 14**).

**Supplementary Table S1.** List of plasmids generated in this study

| Number | Short description | Source | Forward primer(s) used for cloning | Reverse primer(s) used for cloning |
|---|---|---|---|---|
| RJP2303 | pSF3-TOMM20-APEX2-V5 | This study | CCTCCGCGGAATTTCTTAATGATTGTACTGAGAGTTTGG | GGAATAGGAACTTCGCGGCAGTGAGTTAGCTCACTCATTAG |
| RJP2307 | pSF3-TOMM70-APEX2-V5 | This study | CCTCCGCGGAATTTCTTAATGCCACCTGACGTCTAAGAAAC<br><br>AGCAAGATCTGGATAGAGGTTTGGAACTTATC | ACCTCTATCCAGATCTTGCTTCCACTGAAG<br><br>GGAATAGGAACTTCGCGGCAAGAATTCGCTAGCTCTAG |
| RJP2358 | pSF3-FLAG-APEX2-NES | This study | CCTCCGCGGAATTTCTTAATATGGACTACAAGGATGACGAC | GGAATAGGAACTTCGCGGCATTAGTCCAGGGTCAGGCG |
| RJP2359 | pSF3-mito-V5-APEX2 | This study | GCCTCCGCGGAATTCTTAATCACTATAGGGAGACCCAAG | GAATAGGAACTTCGCGGCCACATTAGGCATCAGCAAAC |

**Supplementary Table S2.** List of utilized antibodies

| Reagent | Source | Identifier/Catalog No. |
|---|---|---|
| Anti-TOM20 | Santa Cruz Biotechnology | sc-17764 |
| Anti-TOM40 | Santa Cruz Biotechnology | sc-365467 |
| Anti-TOM70 | Santa Cruz Biotechnology | sc-390545 |
| Anti-GAPDH | Proteintech | 60004-1-Ig |
| Anti-TOM22 | Abcam | ab246862 |
| Anti-APEX2 | Birgit Singer-Krüger (Jansen lab) | - |
| Rabbit IgG Isotype Control | Invitrogen | 02-6102 |
| Anti-TOM40 | Proteintech | 18409-1-AP |
| Anti-V5 | Invitrogen | R96025 |
| Anti-beta-actin | Sigma Aldrich | A1978 |
| Anti-FLAG | Agilent | 200473 |
| Alexa Fluor 488 | Invitrogen | A21206 |
| Neutravidin biotin-binding protein | Invitrogen | A2666 |
| Alexa Fluor 647 | Invitrogen | A20006 |
| Alexa Fluor 555 | Invitrogen | A21422 |
| Alexa Fluor 568 | Invitrogen | A11077 |
| Anti-SYNJ2BP | Proteintech | 15666-1-AP |
| Anti-TOM20 | Proteintech | 11802-1-AP |
